# Genome-Wide Characterization of Zebrafish Endogenous Retroviruses Reveals Unexpected Diversity in Genetic Organizations and Functional Potentials

**DOI:** 10.1128/spectrum.02254-21

**Published:** 2021-12-15

**Authors:** Jun Bai, Zuo-zhen Yang, Hao Li, Yun Hong, Dong-dong Fan, Ai-fu Lin, Li-xin Xiang, Jian-zhong Shao

**Affiliations:** a College of Life Sciences, Key Laboratory for Cell and Gene Engineering of Zhejiang Province, Zhejiang University, Hangzhou, People’s Republic of China; b Laboratory for Marine Biology and Biotechnology, Qingdao National Laboratory for Marine Science and Technology, Qingdao, People’s Republic of China; University of Sussex

**Keywords:** zebrafish, endogenous retrovirus, structure, expression, evolution

## Abstract

Endogenous retroviruses (ERVs) occupy a substantial fraction of mammalian genomes. However, whether ERVs extensively exist in ancient vertebrates remains unexplored. Here, we performed a genome-wide characterization of ERVs in a zebrafish (Danio rerio) model. Approximately 3,315 ERV-like elements (*Dr*ERVs) were identified as Gypsy, Copia, Bel, and class I−III groups. *Dr*ERVs accounted for approximately 2.3% of zebrafish genome and were distributed in all 25 chromosomes, with a remarkable bias on chromosome 4. Gypsy and class I are the two most abundant groups with earlier insertion times. The vast majority of the *Dr*ERVs have varied structural defects. A total of 509 *gag* and 71 *env* genes with coding potentials were detected. The *env*-coding elements were well-characterized and classified into four subgroups. A ERV-E4.8.43-DanRer element shows high similarity with HERV9NC-int in humans and analogous sequences were detected in species spanning from fish to mammals. RNA-seq data showed that hundreds of *Dr*ERVs were expressed in embryos and tissues under physiological conditions, and most of them exhibited stage and tissue specificity. Additionally, 421 *Dr*ERVs showed strong responsiveness to virus infection. A unique group of *Dr*ERVs with immune-relevant genes, such as *fga*, *ddx41, ftr35, igl1c3*, and *tbk1*, instead of intrinsic viral genes were identified. These *Dr*ERVs are regulated by transcriptional factors binding at the long terminal repeats. This study provided a survey of the composition, phylogeny, and potential functions of ERVs in a fish model, which benefits the understanding of the evolutionary history of ERVs from fish to mammals.

**IMPORTANCE** Endogenous retroviruses (ERVs) are relics of past infection that constitute up to 8% of the human genome. Understanding the genetic evolution of the ERV family and the interplay of ERVs and encoded RNAs and proteins with host function has become a new frontier in biology. Fish, as the most primitive vertebrate host for retroviruses, is an indispensable integral part for such investigations. In the present study, we report the genome-wide characterization of ERVs in zebrafish, an attractive model organism of ancient vertebrates from multiple perspectives, including composition, genomic organization, chromosome distribution, classification, phylogeny, insertion time, characterization of *gag* and *env* genes, and expression profiles in embryos and tissues. The result helps uncover the evolutionarily conserved and fish-specific ERVs, as well as the immune-relevant ERVs in response to virus infection. This study demonstrates the previously unrecognized abundance, diversification, and extensive activity of ERVs at the early stage of ERV evolution.

## INTRODUCTION

Endogenous retroviruses (ERVs) are relics of retroviruses that have integrated into host genomic DNA after germ line infection and are thus directly transmissible from parent to offspring across generations (1). ERVs are expanded through cycles of forward and reverse transcription and heritable integration (2). Most ERVs accumulated a large number of mutations, such as insertions, substitutions, recombination, deletions, and partial open reading frame (ORF) loss, under natural selective pressure and thus become non-coding regions (3, 4). A small number of ERVs retain coding ability; however, the vast majority of ERVs are silenced by epigenetic modification (5). ERVs are abundant in the genomes of most jawed vertebrates, and they comprise up to 8% to 10% of human and mouse genomes (4). ERVs have been classified into three major groups in humans and mouse models, including class I–III ERVs, which are related to gammaretroviruses and epsilonretroviruses, betaretroviruses, and spumaretroviruses, respectively (6, 7). Structurally, typical ERVs comprise identical long terminal repeats (LTRs) at both ends and *gag*, *pol*, and *env* genes that respectively encode capsid, polymerase, and envelope-like proteins that resemble those of retroviruses (8).

Although most of the ancient ERVs or elements in human and mouse genomes are defective and do not result in the generation of infectious viruses, hundreds of copies of nondefective ERVs were found to produce functional proteins or non-coding RNAs, which play crucial roles in normal physiologic processes (9, 10). In fact, the expression of human ERVs (HERVs) and mouse ERVs (MERVs) has been implicated in various biological activities, such as gene regulation, epigenetic control, immunomodulation, sexual reproduction and differentiation, RNA interference, and even intercellular RNA transmission (9, 11–16). Perhaps the best examples of ERV-derived functional proteins are Syncytin and Arc, which are crucial regulators in placental morphogenesis and synaptic plasticity that are widely distributed in humans, mice, and other placental animals. The primary structures of Syncytin and Arc are similar to those of retroviral Env and Gag proteins (16–20). In mice, Friend virus susceptibility 1 (Fv1) and 4 (Fv4) are two ERV elements that show high sequence similarity to murine leukemia virus (MuLV); and these two elements are proved to restrict MuLV infection by competitively binding to viral proteins or cellular receptors (21–24). In addition, aberrant activated HERVs were also implicated in many pathological processes, such as tumor development, schizophrenia, reproductive pathology and autoimmune diseases, including multiple sclerosis, systemic lupus erythematosus, type I diabetes, and inflammatory neurologic disorders (25, 26).

Retroviruses exclusively infect vertebrate species (27); fish has been the most primitive host for retroviruses. By phylogenetic analysis between exogenous retroviruses (XRVs) and ERVs, retroviruses were speculated to emerge together with their vertebrate hosts in the ocean (28, 29). Besides the coevolution of retroviruses and host, water–land transmission event was proposed by ERV records, which provide new insight into the virus–host interaction history (27). Thus, fish is integral to understand the evolutionary history of the ERV superfamily. An endogenous retrovirus, referred as ZFERV, was identified in the Tubingen stock of zebrafish (30). This ZFERV has a genome of 11.2 kbp with intact coding regions for *gag*, *pol*, and *env* genes and remains transcriptionally active. ZFERV expression is high in larval and adult zebrafish thymus and minimal in 2 days postfertilization (dpf) embryos (30). Besides, ERVs with coding potential were identified in several other fish species (30–32). The *percom*ORF sequence detected in spiny-rayed fish is believed as one of the most ancient intact Env protein-encoding genes; and the long-term conservation of this homolog indicates its important function during evolution (33). Although preliminary investigations were performed on fish ERVs, a comprehensive understanding of abundant ERVs in fish remains largely limited. Zebrafish (Danio rerio) is a well-established experimental organism for modeling various aspects of physiology and development, given its convenient genetic and embryo manipulation, and fast embryonic growth with typical developmental periods, including zygote, cleavage, blastula, gastrula, segmentation, pharyngula, and hatching stages (34). Zebrafish possesses 25 pairs of chromosomes, among which the chromosome 4 was highly heterochromatic and was presumed to be related with sex determination (35). The well annotated zebrafish genome sequence is immensely informative for extensive comparative genomics and also becomes a valuable tool for phylogenetic and evolutionary research. In the present study, we explored the genome-wide characterization of ERVs and related elements in zebrafish in an attempt to provide a survey of the composition, classification, phylogeny, expression, and functional potential of ERVs in embryo development and adult tissues under physiological and virus infection conditions in an ancient vertebrate organism. This study is anticipated to improve the current understanding of the molecular and functional evolutionary history of ERVs from fish to mammals throughout vertebrate evolution.

## RESULTS

### Genome distribution of *Dr*ERVs.

**FIG 1:**
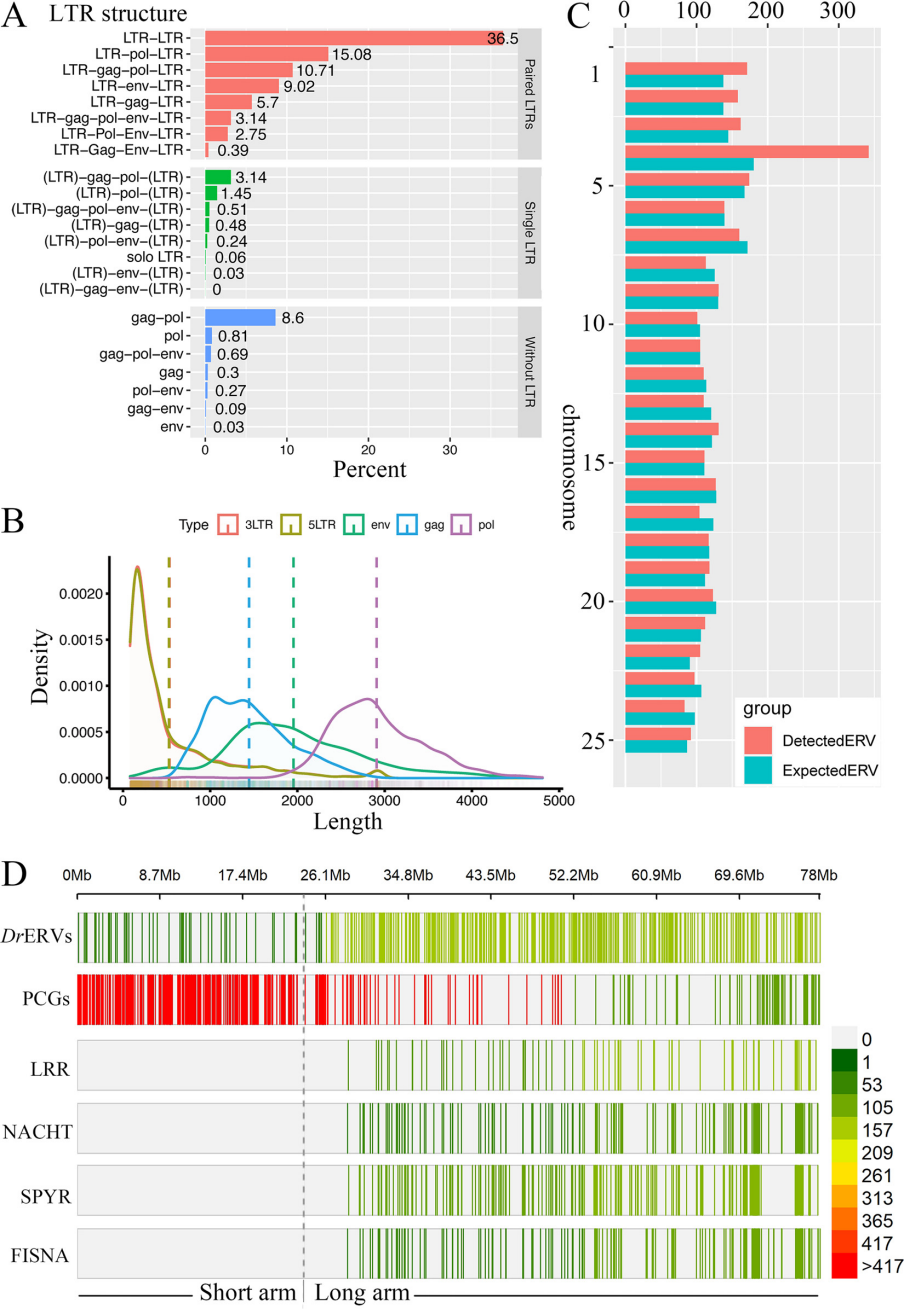
Structural characterization and genome distribution of *Dr*ERVs. (A) Structural elements and proportion statistics of *Dr*ERVs. LTR in parentheses (LTR) represents only single LTR existing at either end. (B) Length statistics of *Dr*ERVs. (C) Chromosome distribution of *Dr*ERVs. The expected ERV number was calculated by multiplying the chromosome length and whole genome average density of *Dr*ERV. The detected ERV number represents the number of actually identified *Dr*ERVs. (D) Distribution of PCGs, *Dr*ERVs, and NLR elements in chromosome 4. Dotted line indicates the boundary between the long arm and the short arm.

A total of 3,315 *Dr*ERV-like elements were identified from zebrafish genome by RetroTector prediction with scores ranging from 250 to 1,247, among which 1,453 have an empirically high score of over 300 (36) (Fig. S1 to S4, Table S1). These *Dr*ERVs account for approximately 2.3% of the zebrafish genome and are distributed in all 25 chromosomes with different numbers. Approximately 3.1% of the *Dr*ERVs possess complete structure with *gag*, *pol*, and *env* genes and LTRs at both ends; whereas the remaining 96.9% of the *Dr*ERVs share partial sequences that are structurally incomplete in varying degrees (Fig. 1A). The most abundant *Dr*ERV structure is the LTR–LTR (36.5%), which means that all the *gag*, *pol*, and *env* genes are lost in this case. Elements with LTRs on both ends account for 83.3% in total and 46.8% when LTR–LTR type is excluded. These outcomes showed the prevalence of *Dr*ERVs with both LTRs in the genome. Among the *Dr*ERVs with recognizable remains of protein-coding genes (PCGs), the LTR–*pol*–LTR accounted for 15.1%. In fact, *pol* is the most detected PCG; hence, the *pol* portions of *Dr*ERVs are retained more frequently than *gag* and *env* portions. The lengths of 5′-LTR and 3′-LTR are comparable and most concentrated in approximately 500 bp (Fig. 1B). The lengths of *gag*, *env*, and *pol* genes are 551 to 4,902 bp, 218 to 4,634 bp, and 709 to 4,810 bp, respectively, and concentrated in 1,000 to 2,000 bp (for *gag* and *env*) and 2,000 to 3,000 bp (for *pol*). The distribution of LTRs and *gag*, *env*, and *pol* genes on positive and negative strands shows no remarkable bias. The number of *Dr*ERVs showed an overall high correlation with the length (*R *= 0.807, *P* <  0.001) and GC content (*R *= 0.797, *P* <  0.001) of zebrafish chromosomes. The correlation between *Dr*ERV abundance and GC content could be associated with high epigenetic modification in *Dr*ERV sequences as observed in other species (37). However, *Dr*ERVs are not always randomly distributed on zebrafish chromosomes. *Dr*ERVs are remarkably enriched in chromosomes 1 and 4 than expected, especially in chromosome 4 (Fig. 1C, Table 1). The densities of *Dr*ERVs located on chromosomes 1 and 4 are 2.87 and 4.38 elements/Mb, which are considerably higher than the average density (2.31 elements/Mb) of *Dr*ERVs distributed on the whole genomes (Table 1). Interestingly, more than 400 duplicated Nod-like receptor (NLR) genes or fragments were observed in zebrafish genome (38, 39). These NLR elements are also highly accumulated on chromosome 4 as detected by the central nucleotide oligomerization domain (NACHT), Fish-specific NACHT associated domain (FISNA), leucine-rich repeat domain (LRR), SPla and the RYanodine Receptor domain (SPRY) independently. We found through comparative analysis that most PCGs are distributed in the short arm of chromosome 4, whereas NLR elements and *Dr*ERVs have a distribution bias on the long arm of chromosome 4 (Fig. 1D). This finding suggests the existence of a close evolutionary correlation and functional association between *Dr*ERVs and NLRs.

**TABLE 1:** Number and density of *Dr*ERVs detected on each zebrafish chromosome

Chromosome no.	Length (Mb)	GC%	Detected ERV no.	Expected ERV no.	Density (ERVs/Mb)	χ^2^ test	χ^2^ test *P* value	
1	59.58	36.4	171	137.6	2.87	8.468	0.004**	↑
2	59.64	36.7	158	137.7	2.65	3.115	0.078	
3	62.63	36.9	162	144.6	2.59	2.182	0.140	
4	78.09	38.4	342	180.3	4.38	153.327	0.000***	↑
5	72.5	36.4	174	167.4	2.4	0.272	0.602	
6	60.27	36.4	139	139.2	2.31	0.000	0.988	
7	74.28	36.7	160	171.5	2.15	0.818	0.366	
8	54.3	36.5	113	125.4	2.08	1.273	0.259	
9	56.46	36.5	131	130.4	2.32	0.003	0.956	
10	45.42	36.6	101	104.9	2.22	0.149	0.700	
11	45.48	36.4	105	105	2.31	0.000	0.998	
12	49.18	36.3	110	113.6	2.24	0.116	0.733	
13	52.19	36.5	110	120.5	2.11	0.953	0.329	
14	52.66	36.6	131	121.6	2.49	0.753	0.385	
15	48.04	36.8	111	110.9	2.31	0.000	0.995	
16	55.27	36.5	127	127.6	2.3	0.003	0.954	
17	53.46	36.6	104	123.5	1.95	3.185	0.074	
18	51.02	36.6	117	117.8	2.29	0.006	0.939	
19	48.45	36.4	118	111.9	2.44	0.346	0.556	
20	55.2	36.6	123	127.5	2.23	0.163	0.686	
21	45.93	36.6	112	106.1	2.44	0.343	0.558	
22	39.13	37	105	90.4	2.68	2.438	0.118	
23	46.22	36.7	97	106.7	2.1	0.918	0.338	
24	42.17	36.3	83	97.4	1.97	2.189	0.139	
25	37.5	36.6	92	86.6	2.45	0.346	0.556	

**, *P*  <  0.01; ***, *P*  <  0.001. ↑, ERV density significantly higher than expected.

### Classification of *Dr*ERVs.

**FIG 2:**
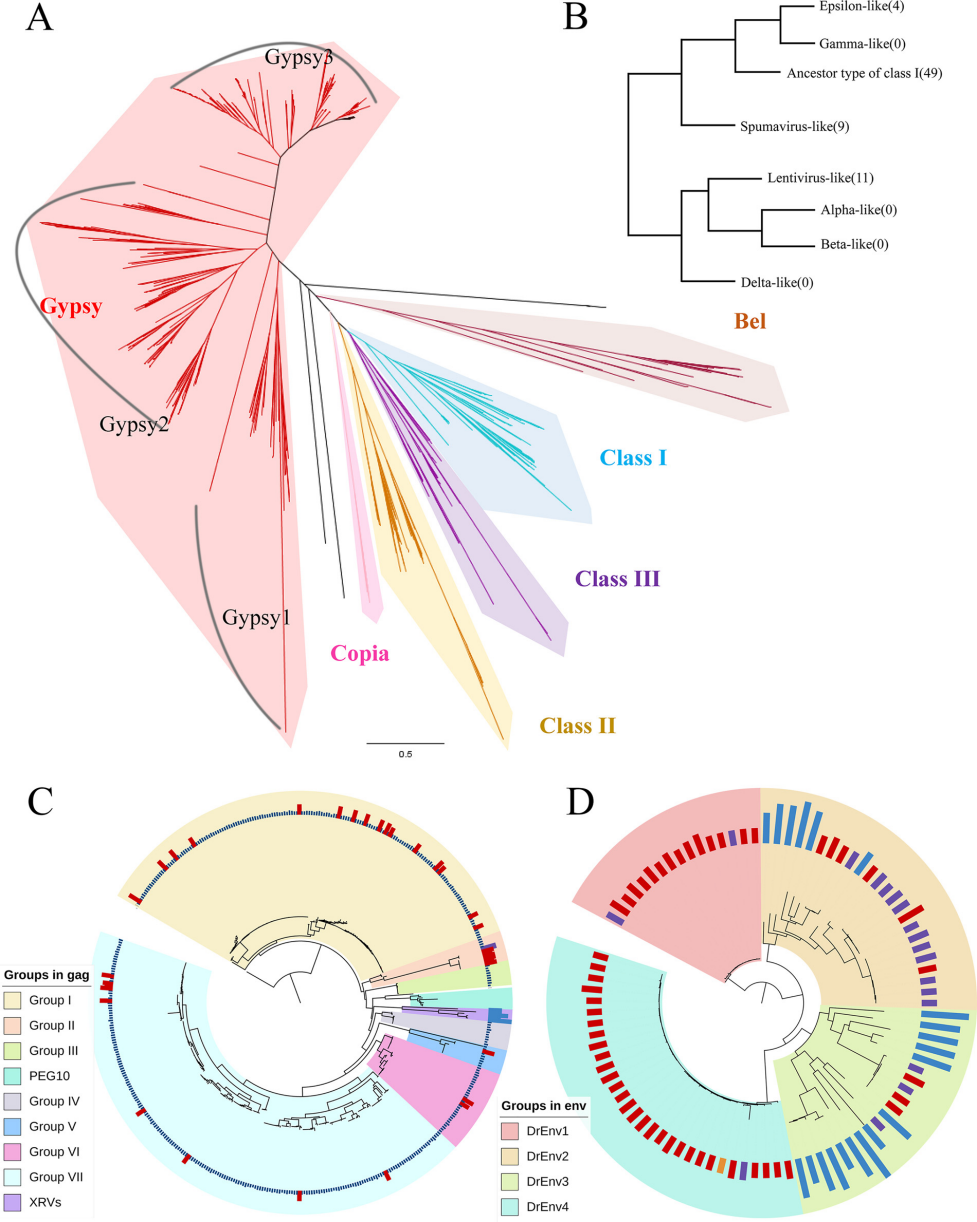
Phylogenetic tree of *Dr*ERV-like elements. (A) Phylogenetic analysis of *Dr*ERVs based on RT region. (B) Schematic diagram of the numbers and relationships between XRV-related *Dr*ERV groups. (C) Phylogenetic tree constructed using retrotrans_gag. (D) Phylogenetic tree constructed using the TLV_coat (HR1–HR2) domain. In (C) and (D), the *Dr*ERVs that have been classified as class I, class III, and gypsy by RT are annotated with purple, orange, and dark blue boxes, respectively; XRVs or ERVs from other species are annotated with light blue boxes; the newly identified *Dr*ERVs are annotated with red boxes.

Given that the concatenated sequences of *Dr*ERVs cannot be properly aligned due to the high sequence diversity, the reverse transcriptase (RT) regions of *pol* genes were applied for *Dr*ERVs classification as described in many other ERV researches (27, 40–43). A phylogenetic tree with 968 *Dr*ERVs containing predictable RT region was constructed (Fig. 2A). These *Dr*ERVs were identified to be Gypsy (830), Bel (41), Copia (6), class I ERV (44), class II ERV (11), and class III ERV (9) (Table S2). Gypsy, Bel, and Copia are sometimes classified as retrotransposon elements, a proposed ancestor of retrovirus that had not acquired the *env* gene during evolution. However, these elements are also considered ERV-like elements in many investigations, because the *env* gene is occasionally found in Gypsy, and infectious Gypsy has been reported (44–46). Therefore, we used the generalized concept of ERV-like elements in this study, and all these elements are considered *Dr*ERVs. Gypsy occupies the largest proportion of *Dr*ERVs and includes three main subgroups (Gypsy1-3) with high divergences. In comparison, Copia and Bel are more convergent compared with Gypsy. Four *Dr*ERVs related to *Snake-head retrovirus* (SnRV) were classified to class III *Dr*ERV, this group is sometimes classified as a separate type (32, 47). As the second most abundant group next to Gypsy, most of class I *Dr*ERVs are more like ancestor type of epsilonretrovirus and gammaretrovirus, and only four are directly related to epsilonretrovirus (Fig. 2B). Only four fish XRVs have been reported, namely, *Walleye epidermal hyperplasia virus* (WEHV), *Walleye dermal sarcoma virus* (WDSV), *Salmon swim bladder sarcoma virus* (SSSV) and SnRV. WEHV, WDSV, and SSSV belong to epsilonretrovirus, which indicating that epsilonretrovirus is prominent in both exogenous and endogenous retroviruses in fish. All 11 class II *Dr*ERVs were clustered with the lentivirus group, and none was clustered with alpha-, beta-, or deltaretroviruses.

Most of the *Dr*ERVs in the genome are structurally incomplete; thus, over 2,000 *Dr*ERVs that lack the RT region were not classified by this method. Although *gag* and *env* genes are usually not used as phylogenetic markers, we applied the relatively conserved retrotrans_gag domain of *gag* and the TLV_coat (HR1–HR2) of *env* to further explore the phylogeny of *Dr*ERVs. Considering that *gag* and *env* are evolutionarily not conserved, the *Dr*ERVs classified by RT region but not exogenous retroviral sequences were used as references in classification. A total of 454 retrotrans_gag domains were identified from 1,119 *gag* genes and form seven subclades. Beyond our expectation, all these 454 elements were related to Gypsy (Fig. 2C), except for ERV-E4.7b.16-DanRer, which has been identified as a class I *Dr*ERV. This outcome could be a result of recombination or transposition. By comparison with the tree constructed by RT region, 34 additional Gypsy-like *Dr*ERVs were newly identified (Table S2). The *env* tree shows that all the 71 elements with TLV_coat (HR1 to HR2) domains are related to class I (Fig. 2D); 51 additional elements were newly identified by this method (Table S2). Thus, 104 class I *Dr*ERVs were identified in total and form the second most abundant group next to Gypsy. All *env* elements were further classified to four groups (named *DrEnv*1 to 4). Among which, *DrEnv*1 and *DrEnv*4 show high degrees of homogeneity, and the elements in these two groups seem to be copies from the same ancestor. By contrast, *DrEnv*2 and *DrEnv*3 show heterogeneity and seem to be correlated with XRVs and mammalian ERVs.

### Insertion time of *Dr*ERVs.

**FIG 3:**
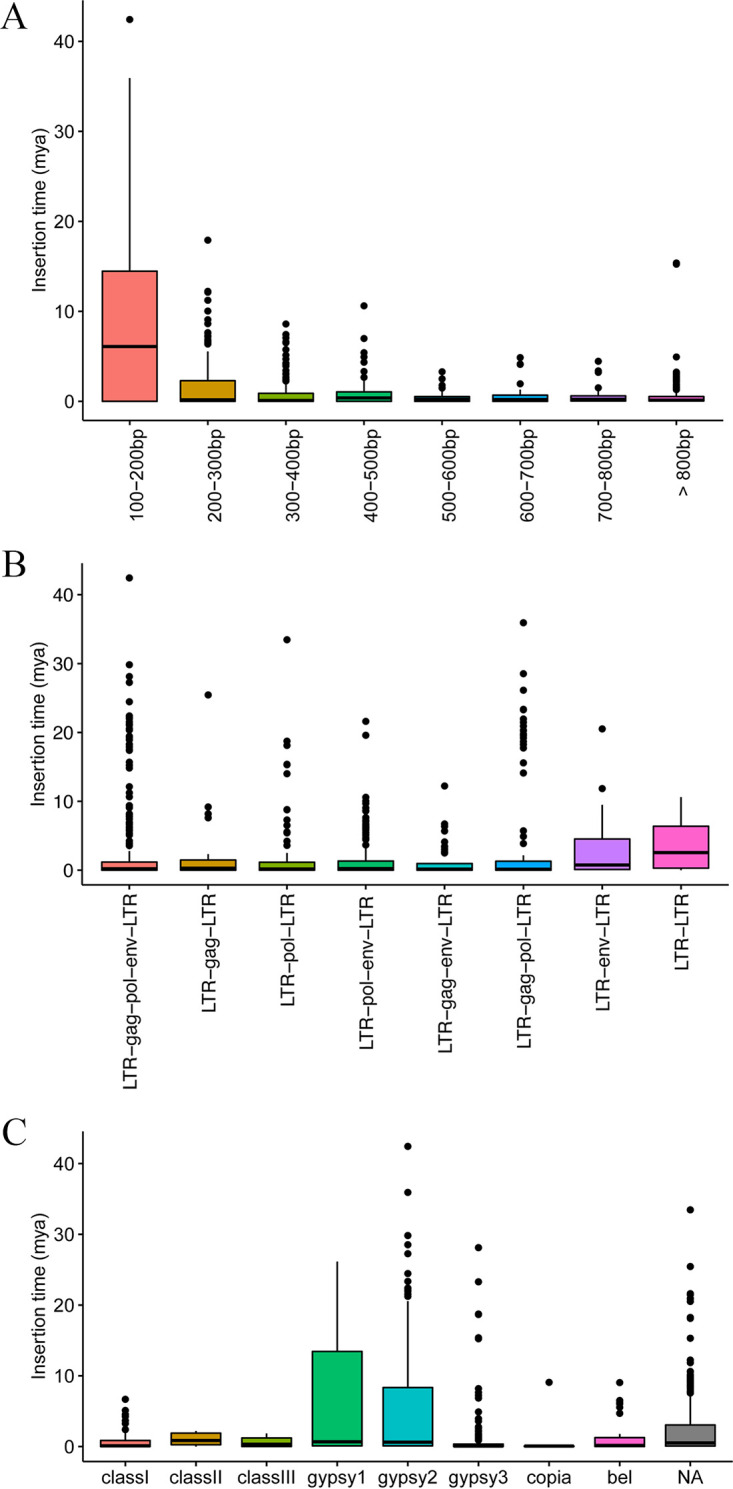
Prediction of insertion times of *Dr*ERVs. (A) The insertion times of *Dr*ERVs with different LTR length. (B) The insertion times of *Dr*ERVs with different structures. (C) The insertion times of *Dr*ERVs in different families.

Identical 5′-LTR and 3′-LTR are believed to start mutating separately at the very beginning of the insertion of retroviruses into host genomes. In this case, the divergence of LTRs at both ends of ERVs are usually used to make a rough estimation of the insertion time. Thus, this method was applied here to reveal the age of *Dr*ERVs, by which a neutral evolutionary rate of 1.46 × 10^−8^ in fish was used (29). A total of 890 *Dr*ERVs with a score of >300 and LTR length of >100 bp were selected for analysis. The result showed that 27.87% (248/890) of the *Dr*ERVs examined possess identical LTRs at both ends. The high proportion of identical LTRs indicates that these elements could be inserted recently or under a strong positive selection pressure. In addition, some *Dr*ERVs might still keep their transposition activity. Overall, the integration times are highly concentrated in 0–1 Mya, with a proportion of 68.54% (610/890) of the *Dr*ERVs. The result implies a recent explosive integration wave in zebrafish. For other intervals, integration waves are observed at about 7 Mya, 9 Mya, 15 Mya, and 17 to 23 Mya (Fig. S5). In addition, we found that the *Dr*ERVs with 100 to 200 bp LTR are much older than others (Fig. 3A). The median insertion time of the former group is 6.10 Mya; whereas it ranges from 0.11 to 0.38 Mya in other groups. This result indicates that the relatively short identifiable LTRs are more likely to reflect the longer-term mutation rather than its inherent characteristics. When focusing on different structures of *Dr*ERVs, the LTR-LTR type shows overall earlier insertion time (median =  2.55 Mya) than other types (median = 0.19–0.75 Mya, Fig. 3B). Actually, the loss of all three PCGs in LTR-LTR type has suggested that it is ancient. Surprisingly, all the *Dr*ERVs integrated over 10 Mya belong to Gypsy (except for the unclassified ones). Gypsy1 possess the highest median insertion time (0.67 Mya) in Gypsy1 to 3; whereas Gypsy2 takes the longest time frame, which possesses the oldest *Dr*ERV (42.43 Mya) in this research (Fig. 3C). In class I–III *Dr*ERVs, class II possess the highest median insertion time (0.86 Mya); whereas the oldest *Dr*ERV (6.68 Mya) in class I–III belongs to class I. The earlier insertion times of Gypsy and class I *Dr*ERVs may have caused the abundance of these two groups.

### Characterization of *gag* genes in *Dr*ERVs.

**FIG 4:**
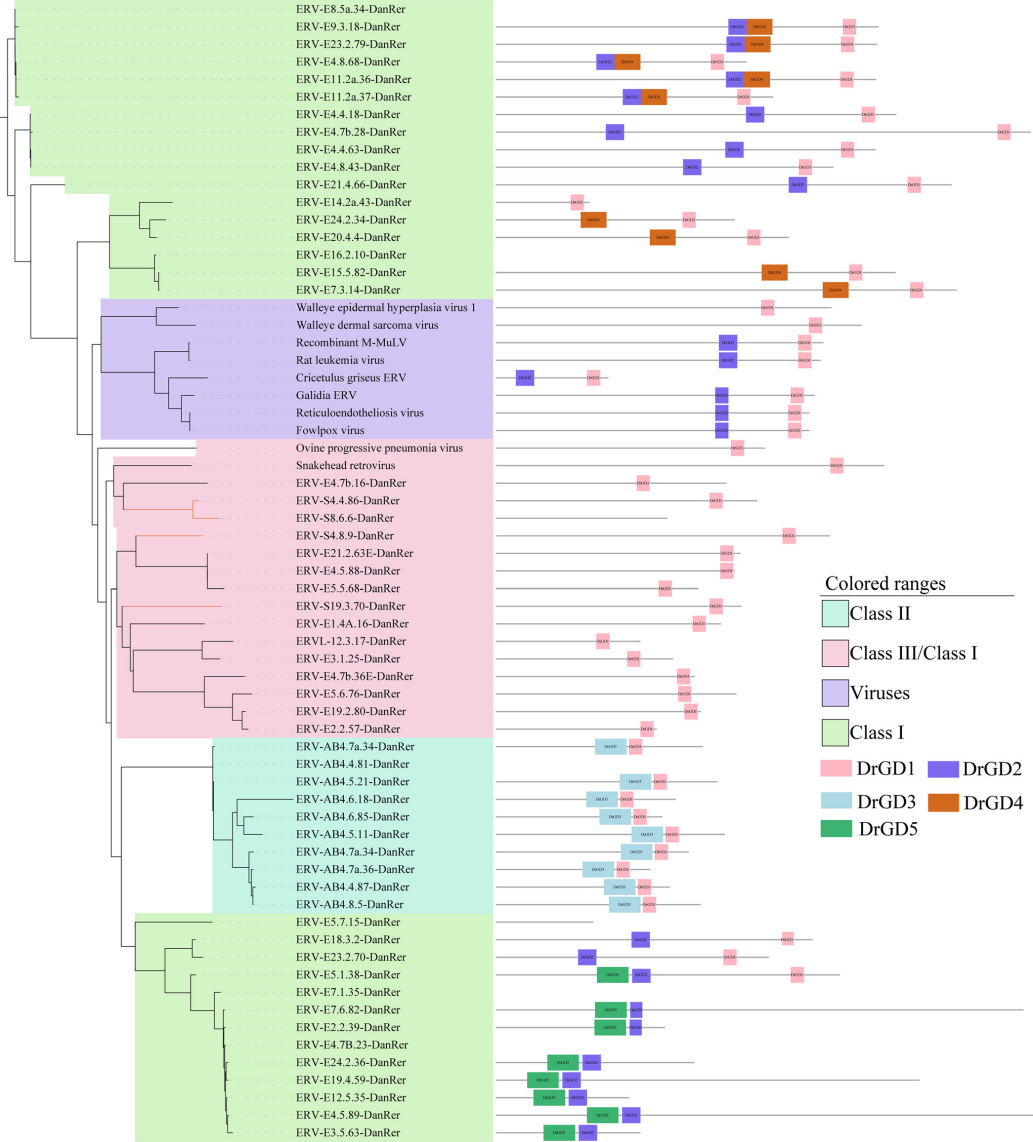
Phylogeny and predicted conserved domains of class I–III *gag* genes without retrotrans_gag domain. The left panel shows the phylogenetic tree constructed with *gag* sequences without predictable retrotrans_gag domain. Eight viruses or ERVs were used as references according to the BLAST results of class I–III *gag* genes without retrotrans_gag domain. The right panel shows the composition of the five predicted conserved domains in each elements. The length ratio between the lines refers to the actual sequence length ratio, and the positions of domains refer to their relative position in the unaligned sequences.

Given that multiple endogenous *gag*- and *env*-derived genes from humans and other species are involved in various diseases, immune responses, and other physiological processes (48–52), we next evaluated the coding potential and gene structure of *gag* and *env* in *Dr*ERVs. According to the phylogenetic analysis of retrotrans_gag, we found that all retrotrans_gag-containing *gag* are Gypsy-like. Besides, several putative *gag*-derived encoding sequences for SRE-ZBP, CTfin51, AW-1, and Number 18 cDNA (SCAN), paraneoplastic Ma antigens (PNMA), and zinc finger protein (Znf) domains were observed at these loci. Among the 1119 *gag* genes, 97 SCAN, 71 PNMA, and 45 Znf domain–encoding sequences were predicted. Interestingly, the predicted domain proteins were all part of or related with zinc finger protein; SCAN and PNMA domains have been proposed to be derived from *gag* (53, 54). We identified 55 ORFs at the typical *gag* positions of class I–III *Dr*ERVs classified by RT to further understand the characteristics of *gag*. Although no domain was predicted in these *gag* loci by the National Center for Biotechnology Information's Conserved Domain Database (CDD), we annotated five potential *gag* domains (assigned as *Dr*GD) by sequence comparison (Fig. S6). In the phylogenetic tree, class I *gag* has two main branches, and *gag* from class II *Dr*ERVs formed an independent clade; however, four class III *gag* formed a mixed clade with some class I *gag* genes, which could be a result of recombination or transposition (Fig. 4). Most XRVs and ERVs from other species, which were selected by BLAST hits of *gag*, formed an independent branch (main virus group); only *Ovine progressive pneumonia virus* (OPPV) and SnRV retrovirus clustered with the mixed clade of class III/class I *Dr*ERVs. We found that *Dr*GD1 is the most representative domain in *Dr*ERVs and kept by all the eight XRVs and ERVs. All the four viruses and two ERVs in the main virus group belong to class I, which comprise two epsilonretroviruses, namely, WEHV-1 and WDSV, and four gammaretroviruses. The epsilonretroviruses possess only *Dr*GD1, but the gammaretroviruses also hold *Dr*GD2, which is a domain found only in the branches of class I *gag*. Although endogenous gammaretrovirus was not identified in zebrafish, this finding suggests that a number of *gag* genes in class I *Dr*ERVs have a close relationship with gammaretroviruses. By contrast, the epsilonretroviruses may have experienced a loss of *Dr*GD2 during evolution. Both OPPV and SnRV belong to class III retrovirus, only *Dr*GD1 was found in these two retrovirus and the clustered class III and class I *gag*. The solo *Dr*GD1 in class III could be a result of the loss of other domains. *Dr*GD3 is a unique domain of class II, *Dr*GD4 and *Dr*GD5 are representative in the top and bottom branches of class III *gag*. These results suggest that the evolution of *gag* is accompanied by obvious domain changes, the zebrafish *gag* genes may be the ancient type that retains more ancestral characteristics. Five domains may be used as markers to identify fish-derived ERVs, especially *Dr*GD1 and *Dr*GD2. Notably, *Dr*GD1 and *Dr*GD2 are retained in XRVs and mammalian ERVs.

### Characterization of *env* genes in *Dr*ERVs.

**FIG 5:**
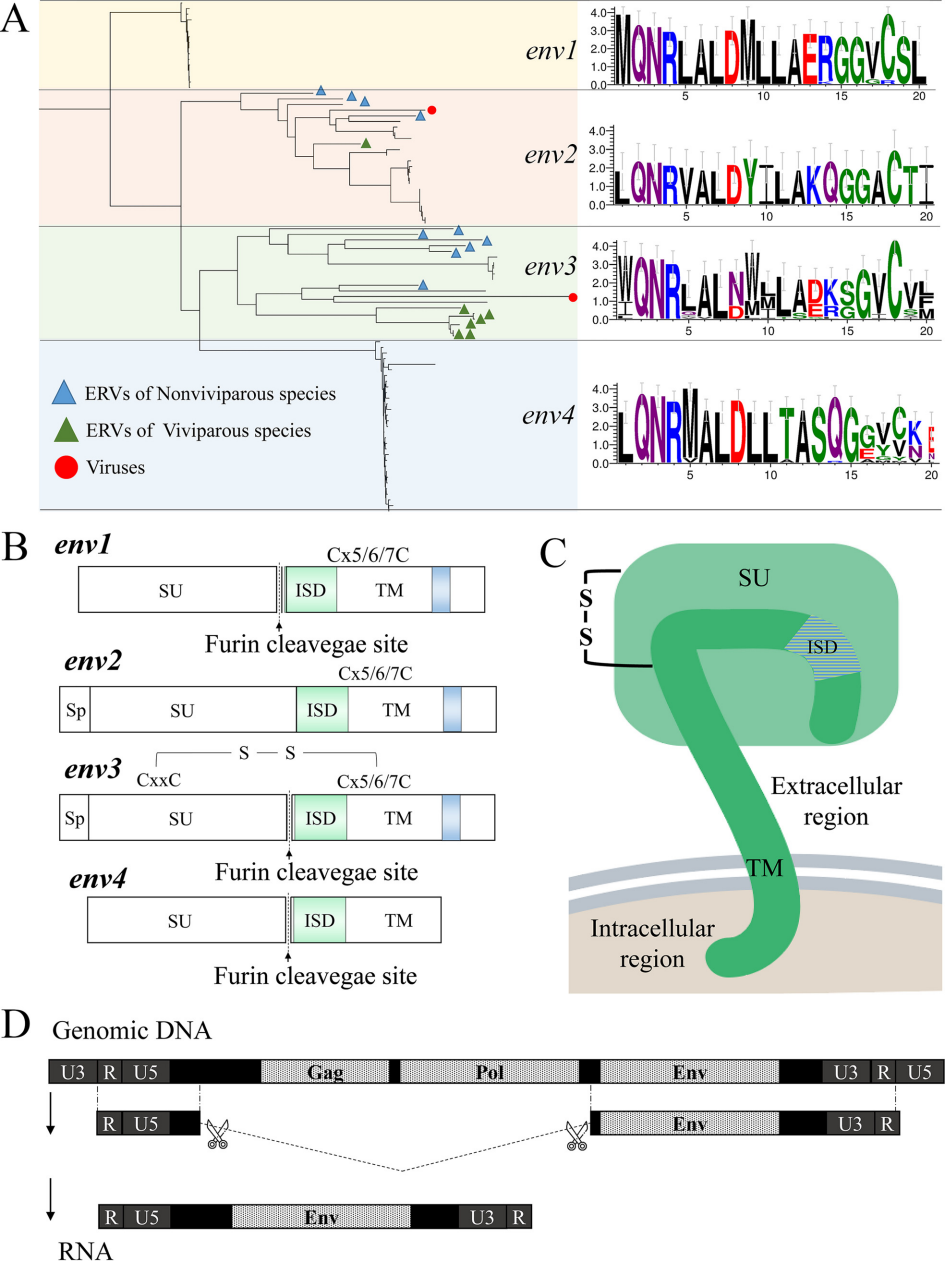
Classification and characteristic of *env* genes in *Dr*ERVs. (A) Phylogenetic tree of 71 *env* genes and diagrams of the ISD of four *env* groups. (B) Annotation of *DrEnv*1–4 protein groups, in which the representative elements are selectively shown. The ERV-E18.4b.4-DanRer of *DrEnv4* does not follow this feature. (C) Pattern diagram of the structure of a representative Env protein located in the cell membrane. (D) Schematic diagram of the transcription and splicing of the *env* gene of ERV-E5.1.38-DanRer.

A total of 71 *env* genes were classified into four groups (*DrEnv*1–4, Fig. 5A). Particularly, *DrEnv*1–3 belong to class I *Dr*ERVs. Most of the *DrEnv*4-containing elements lost the RT region, except for three *Dr*ERVs, namely, ERV-E24.2.36-DanRer (classified to class I), ERV-S3.7.27-DanRer (classified to class III), and ERV-E3.1.25-DanRer (classified to Gypsy). Considering the close phylogenetic relationship between *DrEnv*3 and *DrEnv*4, as well as the classification of ERV-E24.2.36-DanRer, we propose that the exception of ERV-S3.7.27-DanRer and ERV-E3.1.25-DanRer *env* genes comes from the transposition of RT region or *env* genes; therefore, we tend to classify *DrEnv*4 to class I *Dr*ERV as well. The *env* genes in each branch show high sequence similarity. The insertions, deletions, or nonsynonymous mutations are found at various positions in most of the *env* genes. However, none was found to interrupt the putative short ORFs containing the 17 aa immunosuppressive domain (ISD) in *DrEnv*1–4, and some premature stop codons were found right after the ISDs of *DrEnv*4. In addition, the ISD sequences show high consistency in each branch but high heterogeneity across branches (Fig. 5A), among which positions 2 to 4 and 6  to 7 are extremely conserved. The 14th amino acid of ISD was considered to play a principal role in the immunosuppressive function in mammalian Env proteins. Glutamine or arginine/lysine at this site controls the “on” or “off” state of immunosuppressive activity. In *Dr*ERVs, glutamine was found in *DrEnv*2 and *DrEnv*4 at this site, whereas arginine/lysine was found in *DrEnv*1 and *DrEnv*3 at this site.

Four *DrEnv*1, two *DrEnv*2, eight *DrEnv*3, and four *DrEnv*4 possess putative ORFs with more than 1,000 bp in length. The structure of representative *env* genes of each group were analyzed to evaluate gene conservation and the potential function of Env proteins (Fig. 5B). Signal peptides were predicted at the N-terminus of *Dr*Env2 and *Dr*Env3 proteins, indicating the potential of membrane location. However, the signal peptides were absent in *Dr*Env1 and *Dr*Env4 proteins. All of the eight *Dr*Env3 proteins possess a conserved C-X-X-C motif, which was predicted to be involved in surface domain (SU)−transmembrane domain (TM) interaction in the ERVs of other species. However, none was found in the other three *Dr*Env groups. The SU and TM domains of mammalian Env proteins are cleaved by a furin cleavage site with a consensus motif of R/K-X-R/K-R. This motif-encoding sequence was also found in zebrafish *DrEnv*1, *DrEnv*3, and *DrEnv*4 groups, except for *DrEnv*2. The ISD was found in all the four groups, followed by a conserved C-X7-CC motif in *Dr*Env1 and a C-X6-CC motif in *Dr*Env2 and *Dr*Env3. However, this C-X5/6/7-CC motif is absent in *Dr*Env4. The transmembrane region is located at the C-terminus of *Dr*Env1–3 but lost in *Dr*Env4. In conclusion, *Dr*Env4 lost most motifs, but all the eight *env* genes in *DrEnv*3 kept all the coding potential for conserved motifs. The relative position of the motif-encoding sequences in all groups are consistent with *env* genes identified in other species (Fig. 5C) (10, 17, 18). Considering the high integrity of *DrEnv*3, we hope to further evaluate the coding potential and transcription feature of this group. We performed rapid amplification of cDNA ends (RACE) analysis and identified some full-length group members, including an *env*-encoding *Dr*ERV on chromosome 5 (ERV-E5.1.38-DanRer), which is a member of *DrEnv*3. Similar to HERV and other retroviruses, the transcription of ERV-E5.1.38-DanRer starts from the 5′ end of the R region in 5′-LTR and ends at the 3′ end of the R region in 3′-LTR (Fig. 5D). The majority of sequences between 5′-LTR and *env* gene was spliced, including the ORFs of *gag* and *pol*. As a result, the mature transcript retained R and U5 regions at the 5′ end and U3 and R regions at the 3′ end, in which only the ORF of *env* and two noncoding regions at both ends of *env* were kept. This result showed the transcription activity and potential function of the *env* gene in a *DrEnv*3 group member.

### Comparative analysis between *Dr*ERV and HERV.

**FIG 6:**
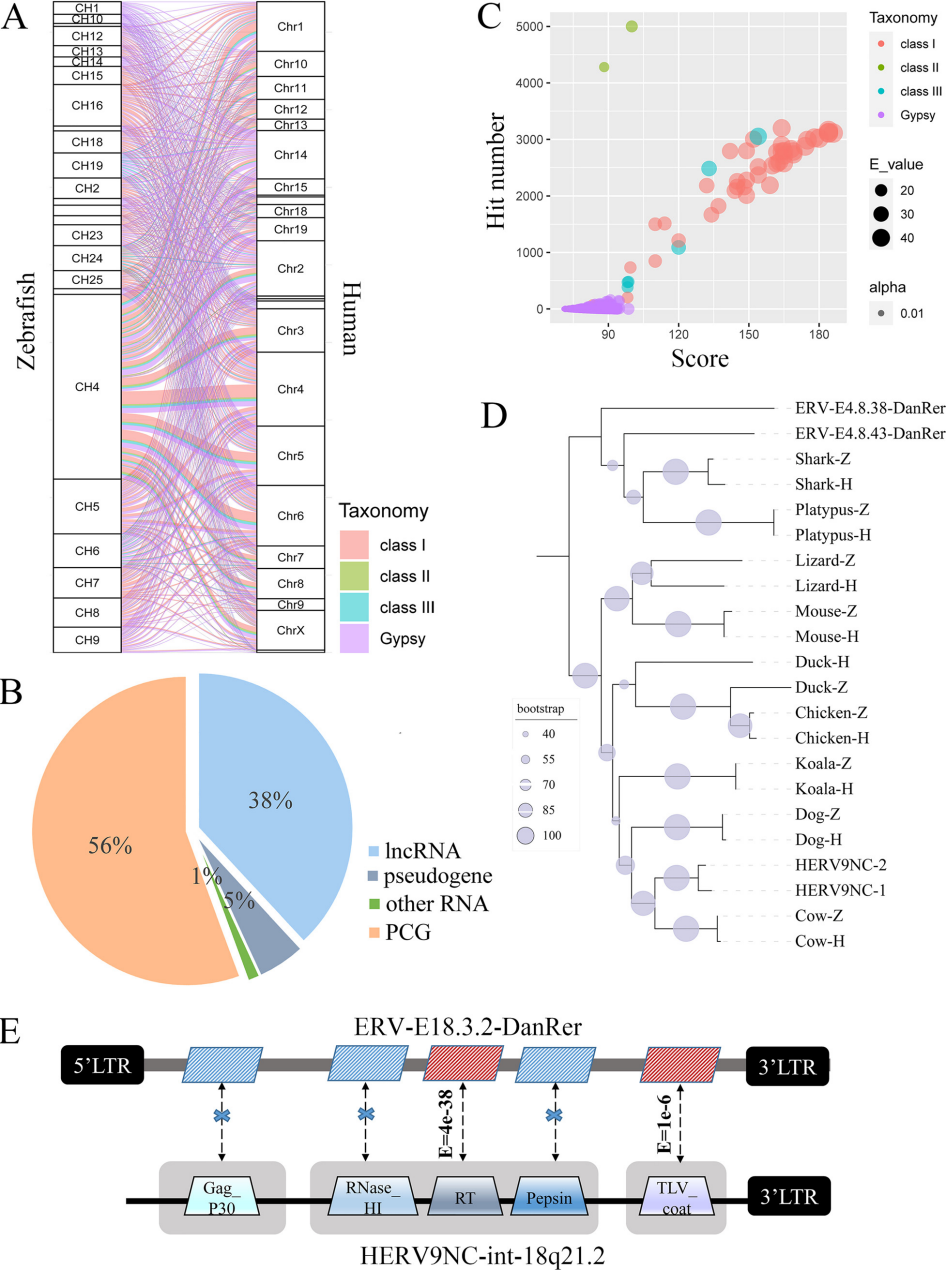
Comparative analysis of *Dr*ERVs and HERVs. (A) Chromosomal distribution of BLAST hits in human genome and query in zebrafish genome. (B) Composition of annotated genes that overlapped with RT BLAST hits. (C) Hit number, score, and E-value of BLAST hits generated by different *Dr*ERV classes. (D) Phylogenetic tree constructed by BLAST hits of HERV9NC-int and ERV-E4.8.43-DanRer in nine species. BLAST hits generated by RT sequences in HERV9NC-int and ERV-E4.8.43-DanRer are annotated by H and Z, respectively. (E) Comparison of the similarity between HERV9NC-int and ERV-E18.3.2-DanRer.

Besides the rapid evolution of the virus itself, the host’s defense mechanism accelerates the mutation of ERVs and makes ERVs highly heterogeneous even among those from the same ancestor. For this reason, understanding the phylogenetic relationship of ERVs across species has received much attention. Here, we performed a comparative analysis between *Dr*ERVs and HERVs to explore the evolutionary history of ERVs from teleost fish to mammals throughout vertebrate evolution. The RT-encoding region of *pol* gene is regarded as the most conserved part of ERVs; therefore, 665 nonredundant RT sequences were used as queries to BLAST the human genome. The highest ERV density was found in zebrafish chromosome 4. In addition, a large number of the BLAST hits (18.1%) in the human genome are generated by the queries of *Dr*ERVs from zebrafish chromosome 4 (Fig. 6A). These results implied that the ERV reservoir of zebrafish chromosome 4 could be primitive and has a profound impact on evolution. Among the 8,919 BLAST hits in the human genome, 2,802 unique targets were found when repeat items were excluded. Most of these targets are intergenic, only 321 targets overlap with annotated genes, among which 56% are PCGs and 38% are lncRNAs (Fig. 6B). We also found that *Dr*ERVs from different taxonomy generate different hit numbers and E-value (Fig. 6C). Although Gypsy comprises the majority of *Dr*ERVs, this group generates only a few hits in the human genome with relatively high E-value. In comparison, most of class I *Dr*ERVs generate a large number of BLAST hits and low E-value in the human genome. Therefore, this group could be quite ancient and may have the most possibility of being evolutionarily preserved ERVs. Furthermore, the BLAST target loci in human genome were examined in ascending order of E-value. Among the top 50 BLAST target sites with the lowest E-values, 25 targets were annotated as different copies of HERV9NC-int; and all these 25 HERV9NC-int elements were generated by class I *Dr*ERVs. We next searched a HERV9NC-int element and its corresponding ERV-E4.8.43-DanRer in other nine species (Fig. 6D). As expected, the BLAST targets with high similarity (E-value <  1e–30) were detected in all the nine species ranging from aquatic fish species to terrestrial mammalian organisms. This outcome implied the potential evolutionary correlation between HERV9NC-int elements and *Dr*ERVs. Next, we compared the other domains of HERV9NC-int and ERV-E4.8.43-DanRer besides RT (Fig. 6E). We found that TLV_coat in the envelope shows a relatively high similarity (E-value = 1e–6), which provides another evidence of the homology of HERV9NC-int and ERV-E4.8.43-DanRer.

In addition, the *env* and *gag* genes of *Dr*ERVs were also applied to BLAST human genome. The target with highest score (E-value =  2e–87) is located in the intron-4 of Ras and Rab interactor 3 (RIN3) of human and chimpanzee, which shows a high similarity with ERV-E19.1.80-DanRer-*env* in a reverse direction (Fig. S7A). The target sequence has a coding potential of a 666 aa protein. The putative TM domain of this protein is substantially more conserved than the SU domain. The TM and SU domains have a genetic distance of 0.8 and 1.2, respectively. Through further analysis, we identified a previously unrecognized HERV element (HERV-14q32.12) at this position (Fig. S8). HERV-14q32.12 contains *gag*, *pol*, and *env* genes and LTRs at both ends. A conserved Gag_p30 domain, a Rve domain, and a TLV_coat encoding sequence were predicted in the *gag*, *pol* and env gene, respectively; however, the usually more conserved RT domain encoding sequence was absent, and *gag* and *pol* were truncated with premature stop codons. The 147 retrotrans_gag domain-containing *gag* genes generate only three nonredundant hits, namely, Retrotransposon Gag-like 1 and 5 and Paternally expressed 10. The *gag* genes that lack retrotrans_gag domain were also used to search the human genome. These elements generate more hits with more substantial E-value, among which ERV-AB4.6.45-DanRer-*gag* and HERVE-int-19p12-*gag* showed highest similarity (E-value =  3e–46) (Fig. S7B).

### Expression of *Dr*ERVs in embryos and adult tissues.

**FIG 7:**
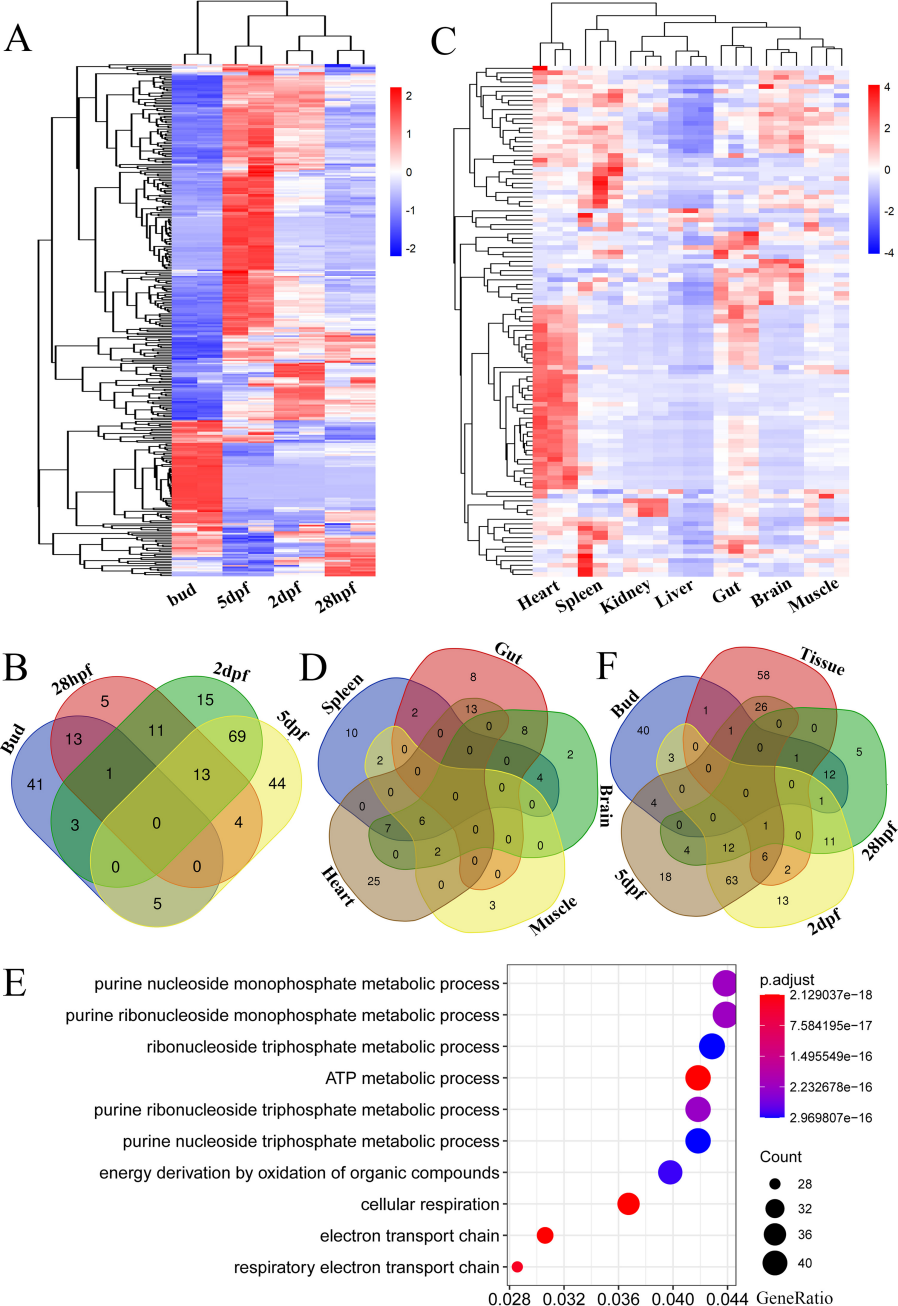
Transcriptional expression analysis of *Dr*ERVs during embryogenesis and in seven tissues. (A) Expression of *Dr*ERVs in four embryo developmental stages. (B) Venn diagram of the overlapping *Dr*ERVs among four embryonic stages. (C) Expression of *Dr*ERVs in seven tissues. (D) Venn diagram of the overlapping *Dr*ERVs among five tissues. Head kidney and liver tissues were excluded because none of the *Dr*ERVs expressed in these two tissues overlapped with the other tissues. (E) GO enrichment analysis of the genes co-expressed with heart-specific *Dr*ERVs. (F) Venn diagram of the overlapping *Dr*ERVs among four embryonic stages and adult tissues. Data from seven tissues were combined as a data set.

The transcriptional expression profile of *Dr*ERVs in zebrafish embryos and adult tissues were analyzed at whole genome-wide level to evaluate the functional activity of *Dr*ERVs in physiological process. The expression pattern of *Dr*ERVs in embryos was analyzed using the RNA-seq data extracted from European Nucleotide Archive. Data of four important developmental stages of segmentation (bud), pharyngula (28-h postfertilization [hpf]), hatching (2-days postfertilization [dpf]) and larval (5 dpf) were chosen. A total of 319 *Dr*ERVs with relatively high expression level (TPM  >  100) were identified at four development stages (Fig. 7A, Table S3). The expression patterns of *Dr*ERVs between bud and 5 dpf stages exhibit substantial difference because the early expressed *Dr*ERVs were hardly expressed in the later period (Fig. 7B). The expression levels of *Dr*ERVs at 28 hpf and 2 dpf stages showed a decrease in bud-specific *Dr*ERVs and an increase of 5 dpf-specific *Dr*ERVs (Fig. 7A). The results indicated the existence of a strict expression regulation between different stage-specific *Dr*ERVs. Compared with the large fraction of LTR–LTR type in the total *Dr*ERVs, a relatively low ratio (18.2%) of this type was highly expressed in embryos, which means that most of the active *Dr*ERVs in embryo have coding potential. Among the *Dr*ERVs expressed in embryos, 10 class I, two class II, and three class III *Dr*ERVs were identified. Among them, six class I and one class III were downregulated at 5 dpf, whereas the others were upregulated. Furthermore, the expression of *DrEnv*1–4 was analyzed. The result showed that some of the *DrEnv*1 and all of the *DrEnv*2 were expressed at the bud stage, whereas most of the rest *DrEnv*1 began to be highly expressed at 5 dpf (Fig. S9). The *DrEnv*3 has broad and diverse expression patterns in embryos, from 28 hpf to 5 dpf, and most of them reached the peak expression at 28 hpf or 2 dpf except ERV-E15.5.82-DanRer, ERV-E4.7b.6E-DanRer, and ERV-E19.1.80-DanRer, which reached the peak expression at 5 dpf. The findings suggest the functional diversity of *DrEnv*3 group members. By contrast, the expression pattern of *DrEnv*4 showed high homogeneity; all of the members reached relatively high expression levels at a late period of embryo development (5 dpf) and exhibited a gradual increase in expression pattern during early development. Given that some previous studies suggested that ERVs could act as regulatory elements for adjacent genes in development, we next analyzed the adjacent genes (within 10 Kb) of bud-specific *Dr*ERVs. Expectedly, 110 known genes were found, 49 of which were expressed at the early stage and downregulated before 5 dpf (Table S4). Interestingly, 22 of the 49 genes were correlated with nucleic acid binding, and nine genes were correlated with ion binding. Further study is needed to elucidate the potential mutual regulation between *Dr*ERVs and their adjacent genes.

Next, the expression pattern of *Dr*ERVs in seven tissues, namely, heart, spleen, head kidney, liver, gut, brain, and muscle, were further evaluated by RNA-seq. Ninety-six highly expressed (FPKM > 1) *Dr*ERVs were identified in the seven tissues (Fig. 7C, Table S5). We found by comparing the seven tissues that only a few *Dr*ERVs are expressed in the liver, whereas considerable *Dr*ERVs are present in other tissues. We found 53 *Dr*ERVs in the heart, 31 in the spleen, four in the head kidney, 31 in the gut, 29 in the brain, and 13 in the muscle. Among these tissue-expressed *Dr*ERVs, 15 *Dr*ERVs were ubiquitously expressed in more than three tissues (Fig. 7D), in which four *Dr*ERVs possess *pol* gene, two *Dr*ERVs possess *gag* and *pol* genes, and nine *Dr*ERVs only have LTR structures at both ends. This result indicates that these LTR–LTR elements are the origin of *Dr*ERV-derived functional noncoding RNAs. By contrast, 47 *Dr*ERVs are tissue specific, that is, 25 *Dr*ERVs are expressed in the heart, 10 in the spleen, four in the head kidney, eight in the gut, two in the brain, and three in the muscle (Fig. 7D). Additionally, 34.0% of the tissue-expressed *Dr*ERVs are class I *Dr*ERVs, 14.4% are Gypsy *Dr*ERVs, and 4.2% are class II and Bel *Dr*ERVs. Notably, the heart is the most active *Dr*ERV-expressing tissue, and most of the heart-expressed *Dr*ERVs (27/53) were class I *Dr*ERVs that contain the *DrEnv*4 genes and account for 87% (27/31) of the total *DrEnv*4-containing *Dr*ERVs identified in zebrafish. This finding suggests that *DrEnv*4 members may play particularly important roles in cardiac function and metabolism. In this respect, we performed a trans-analysis of the 27 heart-specific *DrEnv*4-containing *Dr*ERVs. We found by enrichment analysis that the genes co-expressed with these *DrEnv*4-containing *Dr*ERVs were remarkably enriched in ATP metabolism and cellular respiration (Fig. 7E). This outcome is consistent with the functional characteristics that energy production is particularly prominent in the heart, which is closely associated with mitochondrial function. In addition, three *DrEnv*3-containing *Dr*ERVs (ERV-E19.1.80-DanRer, ERV-E15.5.82-DanRer and ERV-E4.7b.36E-DanRer) were specifically expressed in the brain, and the encoded *Dr*Env3 proteins have the most intact structure of a typical envelope protein. Notably, among the 96 *Dr*ERVs expressed in seven tissues, 39 were also expressed in embryo (Fig. 7F), and 33 of the 39 *Dr*ERVs exhibited an increased expression pattern or started expressing at 5 dpf (Table S6). This result suggests that few *Dr*ERVs expressed in adult tissues were expressed in embryos at early stages (bud and 28 hpf, Fig. 7F). Furthermore, 26 of the 33 *Dr*ERVs have env-coding ability, 24 and two of which were expressed in adult heart and brain. These observations implied the potential functional activity of envelope proteins in adult tissues of zebrafish.

### Expression of *Dr*ERVs in response to *spring viremia of carp virus* infection.

**FIG 8:**
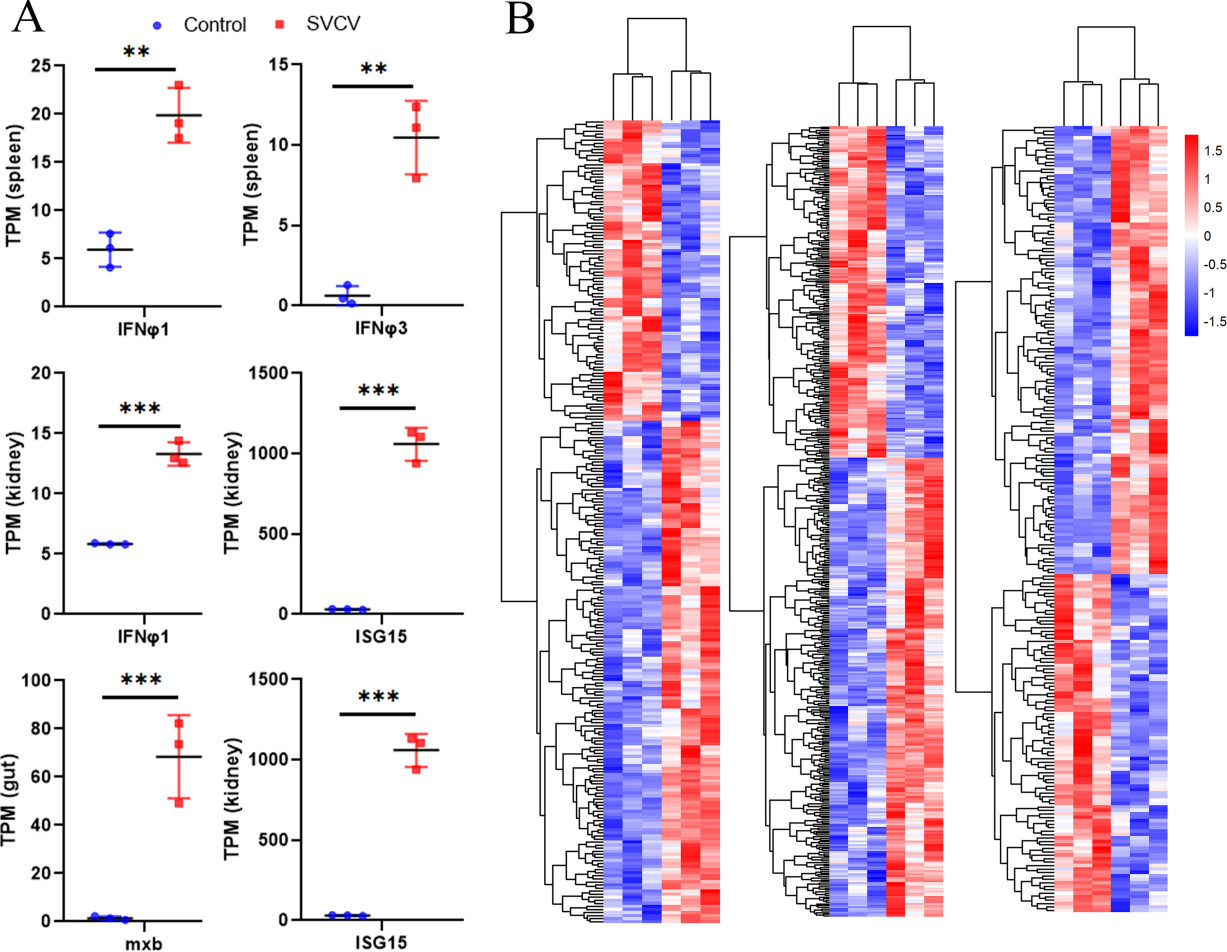
Transcriptional expression analysis of *Dr*ERVs, IFN, and ISG under SVCV stimulation. (A) Upregulated expression of IFN and ISG genes in the spleen, head kidney, and gut tissues of zebrafish in response to SVCV infection (**, *P* < 0.01; ***, *P* < 0.001). (B) Transcriptional expression analysis of *Dr*ERVs in the spleen, head kidney, and gut tissues of zebrafish upon SVCV infection.

We examined the expression profile of *Dr*ERVs in immune-relevant tissues under *spring viremia of carp virus* (SVCV) challenge to explore whether *Dr*ERVs are involved in host antiviral immunity. For this purpose, the head kidney, spleen, and gut, which are the main immune organs that are representatives of central, peripheral, and mucosal immune organs, were selectively examined. The induced-expression of type I interferon (*IFNφ1* and *IFNφ3*) and interferon-stimulated genes (*ISG15* and *mxb*) in the head kidney, spleen, and gut was determined as indicators for the successful initiation of antiviral immunity in response to SVCV infection (Fig. 8A). A total of 192, 130, and 64 *Dr*ERVs were substantially upregulated (Fig. 8B, Table S7). Three and four class I *Dr*ERVs were substantially upregulated in the head kidney and gut, whereas no *Dr*ERVs of the other groups were upregulated. We performed cis- and trans-analyses between *Dr*ERVs and PCGs to preliminarily uncover the potential function of these responsive *Dr*ERVs. Fifteen potential cis-acting genes were found by cis-analysis, and a ERVL-3.4.71-DanRer element was closely correlated with immunoglobulin heavy variable (*ighv*) 4-9 (*R* = 0.95) and *ighv*4-8 (*R* = 0.88). ERVL-3.4.71-DanRer and *ighv*4-9 have a more significant correlation but a longer distance than that between ERVL-3.4.71-DanRer and *ighv*4-8, which indicates a possibility that *Dr*ERVs are correlated with the selection of variable regions in immunoglobulin heavy chain. The trans-acting genes were filtered by correlation coefficient (−0.99 > *R* > 0.99), and a set of 963 genes was generated. A group of G-protein coupled receptors (GPCRs) were found to be functionally correlated with *Dr*ERVs by gene ontology (GO) enrichment analysis (Fig. S10). Given that GPCRs play important roles in various physiological processes, including immune and nervous activities, the discovery of the correlation between *Dr*ERVs and GPCRs may provide new insights into the involvement of *Dr*ERVs in cellular activities through association with GPCRs.

**FIG 9:**
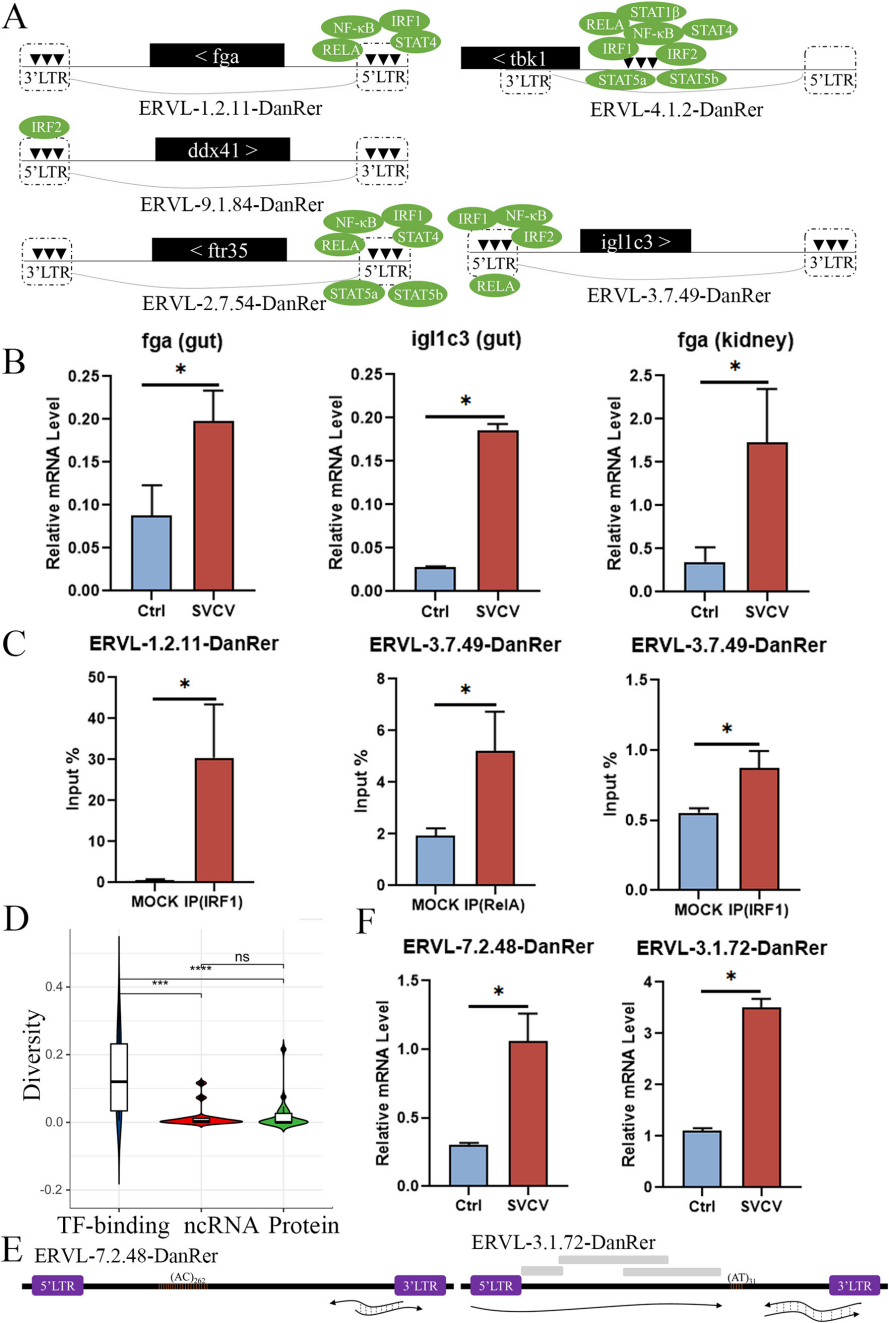
Potential regulatory elements and transcriptional expression analysis of *Dr*ERVs upon SVCV infection. (A) Schematic diagram of the potential TF-binding sites at the LTRs of *Dr*ERVs inserted with different functional genes that were actively expressed in response to SVCV stimulation. These *Dr*ERVs are designated as virus-responsive ERVs (VREs), and their associated functional genes are named as VRE-aid genes. The positional relationship between VREs and VRE-aid genes is shown. (B) Transcriptional expression analysis of two representative VRE-aid genes in head kidney and gut tissues upon SVCV infection. (C) Examination of the TF-binding activity of two representative TFs (IRF1 and RelA) at the LTRs of two VREs by ChIP–qPCR analysis. (D) Comparison of the sequence diversity of LTRs among different VRE types. (E) Schematic diagram of two typical noncoding VREs and the transcripts. The gray box represents the additional LTRs detected inside the *Dr*ERVs. (F) Expression analysis of noncoding VREs upon SVCV stimulation (*, *P* < 0.05; ***, *P* < 0.001; ****, *P* < 0.0001; ns, no significant difference).

We believe that ERVs participate in cellular functions through three ways: providing transcription factor (TF)-binding sites for downstream genes and encoding regulatory noncoding-RNAs (ncRNAs) and functional proteins. We examined the remarkably upregulated immune-relevant genes in response to SVCV infection to verify the first mechanism. We found five potential *Dr*ERV-aid genes, namely, *fga*, *ddx*41, *ftr*35, *igl1c*3, and *tbk*1, which are located inside five *Dr*ERVs, namely, ERVL-1.2.11-DanRer, ERVL-9.1.84-DanRer, ERVL-2.7.54-DanRer, ERVL-3.7.49-DanRer, and ERVL-4.1.2-DanRer. The expression of *fga* and *ddx*41 was upregulated in the gut, and the expression of *ftr*35, *igl1c*3, and *tbk*1 was upregulated in the head kidney and spleen. These genes are potentially regulated by various TFs via association with cis-acting elements (such as promoters and enhancers) in the 5′-LTRs and/or 3′-LTRs of the five *Dr*ERVs. The binding sites of RelA, STAT4, NF-κ B, and IRF1 were found in the 5′-LTR of ERVL-1.2.11-DanRer, which is distributed upstream of the *fga* gene. An IRF2 binding site was found in the 5′-LTR of ERVL-9.1.84-DanRer, which is located upstream of the *ddx*41 gene. The binding sites of STAT4, STAT5a, STAT5b, IRF1, RelA, and NF-κB were found at the 5′-LTR of ERVL-2.7.54-DanRer, which is distributed upstream of the *ftr*35 gene. RelA, IRF1, IRF2, and NF-κB binding sites were located at the 5′-LTR of ERVL-3.7.49-DanRer, which is upstream of the *igl1c*3 gene. Interestingly, numerous binding sites for STAT4, STAT5a, STAT5b, STAT1β, NF-κB, RelA, IRF1, and IRF2 were found at the non-coding region between the 5′-LTR and 3′-LTR of ERVL-4.1.2-DanRer, which is distributed upstream of the *tbk*1 gene (Fig. 9A). The association of IRF1 and RelA with the LTR upstream *fga* and *igl1c*3 genes in ERVL-1.2.11-DanRer and ERVL-3.7.49-DanRer were selectively examined by chromatin immunoprecipitation (ChIP) assay to confirm the binding activity of TFs to the LTRs of *Dr*ERVs. We initially verified the upregulated expression of *fga* and *igl1c*3 in gut and head kidney tissues under SVCV infection (Fig. 9B). ChIP analysis showed that IRF1 and RelA are considerably enriched at the desired LTR regions as expected (Fig. 9C). Thus, the LTRs of *Dr*ERVs inserted with cellular genes could facilitate gene expression by providing various TF-binding sites. Consistent with this notion, the 5′- and 3′-LTRs of these *Dr*ERVs showed higher diversity than those of other *Dr*ERVs (*P*  <  0.01), such as *Dr*ERVs that encode ncRNAs and PCGs (Fig. 9D).

We examined virus-responsive *Dr*ERV elements with non-coding ability, especially those located at intergenic regions, to identify *Dr*ERV-derived ncRNAs. The result showed that 14 *Dr*ERVs strongly expressed ncRNAs in three tissues upon SVCV infection (Table S8). Among them, ERVL-7.2.48-DanRer and ERVL-3.1.72-DanRer were bidirectionally expressed, with a potential to form double-stranded RNA (dsRNA) (Fig. 9E). ERVL-7.2.48-DanRer and ERVL-3.1.72-DanRer showed similar transcription patterns. The transcription of positive strands started upstream of 3′-LTR and ended at the middle of 3′-LTR, whereas the transcription of negative strands occurred exactly in an opposite way. Besides, ERVL-3.1.72-DanRer has another transcript, whose transcription started from the beginning of 5′-LTR. This transcript does not overlap with the potential dsRNA transcript mentioned above. In addition, simple sequence repeat (SSR) elements were detected upstream of ERVL-7.2.48-DanRer and ERVL-3.1.72-DanRer with (AC)_262_ for ERVL-7.2.48-DanRer and (AT)_31_ for ERVL-3.1.72-DanRer. These SSR elements may potentially participate in transcription regulation. Pattern recognition receptors, such as RIG-I and MDA5, can recognize cytoplasmic dsRNAs under virus infection. The dsRNA transcripts derived from ERVL-7.2.48-DanRer and ERVL-3.1.72-DanRer were remarkably upregulated under SVCV stimulation (Fig. 9F). Thus, we proposed that these two *Dr*ERV-derived dsRNAs could facilitate the activation of innate immunity as virus mimicry.

Finally, we found that 41 *Dr*ERVs with protein-coding potential were regulated in the spleen, kidney, and gut tissues in response to SVCV infection (Table S9). Almost all the 41 protein-coding *Dr*ERVs potentially encode entire or partial Pol; 12 of these *Dr*ERVs encode Gag, and two of these *Dr*ERVs encode Env. Both of the two Env proteins belong to the *DrEnv3* group. Besides, five of the 41 *Dr*ERVs encode a SCAN domain-containing protein. The SCAN domain is usually found in zinc-finger proteins and thought to be related to transcription regulation. The sequences that encode the SCAN domain-containing proteins are located between 5′-LTRs and *pol* genes in the five *Dr*ERVs; such a genetic locus is similar to that of *gag* gene, and this domain is proposed to be derived from gag (53).

## DISCUSSION

We systematically identified ERVs in a zebrafish model to understand the origin, evolution, and potential function of ERVs in an ancient vertebrate. Approximately 3,315 *Dr*ERV elements were identified from all the 25 chromosomes of zebrafish and accounted for 2.3% of the zebrafish genome. The vast majority of the *Dr*ERVs are incomplete in structure in varying degrees and the LTR-LTR elements are the most abundant class of *Dr*ERVs. This finding suggests that most *Dr*ERVs are deficient probably because of the autonomous suppression by the host or homologous recombination (1). The *Dr*ERV elements were classified into Gypsy, Bel, Copia, and class I–III groups. Gypsy occupies the largest proportion of *Dr*ERVs with high divergences. The oldest *Dr*ERV, which belongs to Gypsy, was predicted to be integrated into zebrafish genome over 40 Mya. Besides Gypsy, class I *Dr*ERVs were the most abundant group in zebrafish. This group was predicted to be inserted into the genome much older than classes II and III. The early insertion time of class I *Dr*ERVs may cause the abundance of this group. Despite its prevalence, almost all of the class I *Dr*ERVs are an ancestor type of gamma- and epsilon-like retroviruses; only four are directly related to epsilonretroviruses. These ancient class I *Dr*ERVs may promote the monophyletic origination of gammaretroviruses and epsilonretroviruses and provide an epitome of the ancestral features of class I retroviruses. In addition, a total of 11 *Dr*ERVs were clustered with exogenous lentiviruses in *pol*-based phylogenetic tree; however, the regions of *gag* and *env* genes between these *Dr*ERVs and exogenous lentiviruses cannot be properly aligned due to the high sequence diversity. Given that endogenous lentiviruses have not been detected before, the existence of endogenous lentiviruses in fish remains to be further clarified by developing new strategies. In this respect, an improved method for phylogenomics of gammaretroviruses can provide a valuable reference for our research (47, 55). In the prediction of insertion time, we found that considerable *Dr*ERVs possess identical LTR elements at both ends. The cause of this phenomenon could be due to the newly insertion event. Another explanation is that these elements are under strong purifying selection and are therefore involved in physiological events. In addition, these LTRs are theoretically capable of transposition. Retrotector has been designed with algorithms that recognize some retrovirus signature (including LTRs) and their respective distance. It is worth noting that RetroTector does not properly assess solo LTRs, thus this highly defective type of *Dr*ERV is almost absent in this research. Additionally, the accurate prediction of LTRs is still challenging due to the high divergence. Although the SweepDNA and LTRID modules in RetroTector use various strategies to avoid false positive identification of LTRs, we believe that there may still be a certain misrecognition rate at the present stage. Clarification on these issues also depends on methodological innovation in the future.

Interestingly, *Dr*ERVs have a remarkable distribution bias on chromosome 4, especially on the long arm of this chromosome. ERVs are highly enriched on sex-determination chromosomes in humans and other mammalian species, and some previous studies have suggested that chromosome 4 might be related with sex determination in zebrafish (35, 56); thus, our findings may provide new insights into the hypothesis that chromosome 4 links sex determination in zebrafish based on the ERV distribution bias. Additionally, quite a few NLR element-encoding genes densely accumulated on the long arm of chromosome 4. Considering that active ERVs have transposition ability, these NLR-coding sequences may be amplified and fragmented by the surrounding *Dr*ERVs and seem to be isolated from other PCGs in the short arm. This occurrence makes the long arm of chromosome 4 entirely heterochromatic and these NLR elements are under precise controls. The NLR family is an integral part of innate immunity, but we are not clear whether the stacked *Dr*ERVs and NLRs in the long arm region is a result of the game of the host and the intruder. However, the transposition ability is very likely to be the driving force of the duplication of NLRs. The evolutionary correlation and functional interplay among *Dr*ERVs, sex determination, and innate immunity are an interesting topic that remains to be further explained as a whole.

*Gag* and *env* genes are recognized as multifunctional regardless of physiological or pathological conditions; therefore, we next focused on these two genes. The retrotrans_gag is the only characteristic domain found in zebrafish *gag* elements. However, all detected retrotrans_gag domains belong to *gag* in Gypsy. This finding indicates that this domain could originate from Gypsy. In this case, no domain could be predicted by CDD in the *gag* of class I–III *Dr*ERVs. Many long ORFs were found in these *gag* position of class I–III *Dr*ERVs; thus, we aligned and recognized five potential *gag* domains (*Dr*GD1–5) in these elements. *Dr*GD1 and *Dr*GD2 were detected in *Dr*ERVs, XRVs, and mammalian ERVs. Therefore, these two domains are relatively conserved in evolution and could become new functional cores. In addition, these domains could also be applied as new markers for ERV identification in lower vertebrates. Since the discovery of syncytin in mammals, endogenous Env protein has received much attention (57, 58). All the 71 newly identified *env* in zebrafish belong to class I *Dr*ERVs, because no *env* was found in the other groups. Retroviruses were generally proposed to have originated from transposons by acquiring *env*-like genes and thus gained interhost transposition ability, although some LTR retrotransposons seem to be originated from retroviruses via passive loss of the *env* genes (59, 60). Based on this theory, we could speculate that the *env* genes in class I retroviruses were captured in fish genome or even earlier. The abundance and early insertion time of class I *Dr*ERVs are also in accordance with this hypothesis. In humans, gamma- and epsilon-related HERVs have been detected. Different from those of zebrafish, gamma-related HERVs are the most abundant class I HERVs (7). However, only the ancestors of class I and epsilon related *Dr*ERVs were detected in zebrafish, and no gamma-related *Dr*ERVs were found. Coincidentally, almost all the known fish XRVs belong to epsilonretrovirus, although most fish XRVs may go undetected. This finding hints the profound effect of epsilonretrovirus on fish genome, or reverse.

More than 88% of the BLAST hits are located in the intergenic regions when the RT elements of *Dr*ERVs were used to BLAST the human genome. This finding indicates that ERV elements are an important resource of non-coding regions in vertebrate genome. A quite large number of BLAST hits in the human genome were generated by *Dr*ERVs on zebrafish chromosome 4. This result proved again that the *Dr*ERVs at the long arm of chromosome 4 could be functional and seem to be evolutionarily conserved and even vital in species evolution. We found through the comparison between *Dr*ERVs and HERVs that HERV9NC-int has the highest homology to *Dr*ERVs, particularly ERV-E4.8.43-DanRer. This HERV possesses coding potential for *gag*, *pol*, and *env* genes and is responsive to HIV infection (61); thus, it seems to be an undiscovered important HERV in the human genome. This finding is consistent with our usual understanding that elements with vital functions are evolutionarily conserved. Numerous ERVs with high identity were found by searching HERV9NC-int and ERV-E4.8.43-DanRer in other species. Phylogenetic analysis showed that the BLAST hits from the same species are clustered together, regardless if the hits were generated by HERV9NC-int or ERV-E4.8.43-DanRer. In addition, when the *gag* and *env* elements of *Dr*ERVs were applied to BLAST the human genome, hits with high E-value (3e-46 and 2e-87, respectively) were detected. The results suggest that ERV elements could also be preserved during vertebrate evolution like normal genes and these ERV elements are very likely to play fundamental roles in biological activities. Besides that, these analogous elements are also possibly derived from large-scale cross-species transmissions.

A total of 665 *Dr*ERVs were actively expressed in embryos and adult tissues under physiological and viral infection conditions. These *Dr*ERVs account for 20.06% of the total *Dr*ERVs in zebrafish. The results suggest the extensively involvement of *Dr*ERVs in the life activities of zebrafish, including embryonic development, cellular metabolisms, tissue homeostasis, and immune responses. Among the 665 actively expressed *Dr*ERVs, 319 *Dr*ERVs were detected in embryos at four developmental stages, 96 *Dr*ERVs were found in seven adult tissues under normal physiological conditions, and 421 *Dr*ERVs were strongly induced in three tissues upon viral infection. The majority of embryo-specific *Dr*ERVs were differentially expressed at the four developmental stages, namely, bud, 28 hpf, 2 dpf, and 5 dpf. These *Dr*ERVs show distinct stage-specific transcriptional patterns during zebrafish development. The stage-specific genes or elements are closely related to cell lineage specification for ongoing development; therefore, the identification of lineage-specific *Dr*ERVs from each developmental stage needs to be further explored. A clarification on this issue would provide new insights into the mechanisms underlying ERV-based regulation of embryonic development. The 96 tissue-expressed *Dr*ERVs were mostly enriched in the heart, followed by the spleen, gut, brain, muscle, head kidney, and liver. Among these 96 *Dr*ERVs, 15 *Dr*ERVs were ubiquitously expressed in different tissues, whereas 48 *Dr*ERVs were expressed in specific tissues. The tissue-specific *Dr*ERVs were most abundant in the heart, followed by spleen, gut, head kidney, muscle, and liver. The tissue-expressed *Dr*ERVs exhibited a wider encoding potential for Env, Pol, and Gag proteins and noncoding RNAs. This encoding potential meets the requirement for the functional diversification of different tissues. In addition, 38 *Dr*ERVs expressed in adult tissues were also transcribed in embryos, and most of them were detected at 5 dpf. This finding suggests that these *Dr*ERVs play important roles in fundamental cellular activities throughout the lifetime of zebrafish from the late-developmental embryos to adult tissues. Notably, considerable numbers of embryo- and tissue-expressed *Dr*ERVs belong to class I *Dr*ERVs. Hence, this type of *Dr*ERV has stronger functional activities in cellular development and metabolism than those of other types.

Importantly, 421 *Dr*ERVs were remarkably induced in head kidney, spleen, and gut tissues in response to SVCV infection. Most of these *Dr*ERVs were not transcribed in tissues at steady state under normal conditions without SVCV stimulation; hence, they are viral-responsive *Dr*ERVs, which are crucial for antiviral immunity in zebrafish. Given that the head kidney, spleen, and gut are three major immune-relevant tissues that represent the central, peripheral, and mucosal immune systems in fish, the vastly induced expression of *Dr*ERVs in these tissues suggests that *Dr*ERVs play extensive roles in a wide spectrum of immunities, which potentially rang from the activities in the early hemopoietic regulation, proliferation, and differentiation of immune cells in systemic immunity to local defense reactions in mucosal immunity. The correlation between ERVs and exogenous viruses has long been a challenging topic that remains to be explored. Some previous investigations have focused on the interplay between ERVs and XRVs, such as HIV, spleen necrosis virus, MuLV, and friend virus (62). However, the association between ERVs and a non-retroviral virus remains poorly understood. In the present study, we demonstrated that numerous *Dr*ERVs are highly responsive to SVCV, a negative-stranded RNA virus and a member of family *Rhabdoviridae*. Thus, zebrafish is expected to be an attractive model organism for studying the complex relationships among ERVs, exogenous invading viruses, and host immunity. Such investigations may include the identification of the regulators (such as stimulators, restriction factors, and interactomes) of ERV expression, the sensing and control of ERVs by innate-immune signaling pathways and underlying mechanisms, the regulation of innate immunity by ERVs, the evolutionary history of ERVs, and the co-evolution mechanism between ERVs and host immunity.

The ERV elements occur in four broad classes in humans and mouse models, including elements that are relatively intact with potentially infectious retrovirus members; elements that lack partial coding sequences, typically *env*, but potentially autonomous; nonautonomous elements that lack coding sequences but retain essential *cis*-acting sequences for transcription, packaging, and primer binding; and solo LTR products of recombination between LTRs and the associated loss of the internal domain and one LTR copy (2, 3, 63). All these four ERV classes were identified in zebrafish and the most abundant class is that of LTR-LTR elements. Importantly, a fifth class of ERVs was identified in zebrafish. In the fifth class, the internal viral elements within the LTR ends are completely replaced by some cellular functional genes, especially immune-relevant genes, such as *fga*, *ddx41*, *ftr35*, *igl1c3*, and *tbk1*. This finding reflects the structural and functional bias of *Dr*ERVs that were repurposed for host gene expression under strong selection pressure during evolution. Architecturally, the LTR structures are a rich source of transcriptional regulatory *cis*-elements, which provide numerous TF-binding sites in the promoter and enhancer sequences of LTRs. For example, the LTRs with *fga*, *ddx41*, *ftr35*, *igl1c3*, and *tbk1* genes in zebrafish contain perfect or nearly perfect binding sites for the RelA, IRF1, IRF2, NF-κB, STAT1β, STAT4, STAT5a, and STAT5b TFs. Thus, this class of *Dr*ERVs may potentially mediate an immune transcription network in response to viral infection. In addition, we noticed that the heavy chain (*ighv*4-8/9) and light chain (*igl1c*3) of zebrafish immunoglobin-coding sequences are closely correlated with *Dr*ERVs; and a considerable number of *Dr*ERVs regulate the expression of adjacent genes. These observations suggest the long-distance modulatory effect of *Dr*ERVs on remote target genes probably through regulatory elements, such as enhancers in LTRs. Thus, the vast majority of LTR structures in zebrafish genome may provide a large reservoir of regulatory elements for host utilization in various cellular activities and even long-distance genomic recombination, which leads to increased mutation frequency and thus contributes to species evolution (64). The regulatory elements included in LTRs seem to be expandable with evolving host control over invading species, because increasing number of LTR elements were emerged in humans and other mammals (5, 7, 65). In addition, LTRs are known as the focus of epigenetic silencing. In this case, the flanking LTRs of functional genes or elements could undergo reprogramming by epigenetic modifications from repressive modules to active status. Hence, the LTR elements of *Dr*ERVs would become an attractive model platform for understanding the mechanisms underlying epigenetic regulation at genome-wide LTR structural levels.

In conclusion, we comprehensively analyzed the composition, phylogeny, and potential functions of ERVs in zebrafish, an attractive model organism of ancient vertebrates, from multiple perspectives. The results may provide a solid foundation for further investigation on the molecular origin, genetic shift, and functional evolution of vertebrate ERV family from fish to mammals. The ubiquitous existence and high fraction of ERVs in vertebrates determine their important position in the genome, but at present, ERV research still needs to be expounded.

## MATERIALS AND METHODS

### Experimental fish and virus.

Wild-type AB zebrafish (Danio rerio) were bred and maintained in circulating water at 28°C under standard conditions as previously described (66, 67). Only healthy fish, as determined by general appearance and activity level, were used. Zebrafish embryos were collected by natural spawning and kept at 28.5°C in incubator with a 14:10-h light/dark photoperiod. All animal care and experimental procedures were approved by the Committee on Animal Care and Use and the Committee on the Ethic of Animal Experiments of Zhejiang University. SVCV was a gift from Prof. Yibing Zhang (Institute of Hydrobiology, Chinese Academy of Sciences) and propagated in epithelioma papulosum cyprini (EPC) cells as previously described (68).

### Detection of *Dr*ERVs in the genome.

Sequences of 25 zebrafish chromosomes (GRCz11) were downloaded from NCBI Genome database (https://www.ncbi.nlm.nih.gov/genome/?term=zebrafish). The sequences of each chromosome were segmented into 9 Mb fragments with 2 Kb overlapping regions between the fragments and submitted to RetroTector (http://retrotector.neuro.uu.se/) for prediction of *Dr*ERVs with default settings. RetroTector contains three modules for the recognition of candidate LTRs, the recognition of retroviral conserved motifs that meet distance thresholds and the reconstruction of original protein sequences. In addition, RetroTector can give a variety of possible ORFs and score values based on how well the sequences match the infectious retroviruses (36).

### Nomenclature of *Dr*ERVs.

*Dr*ERVs are named following the rules described previously (69). Briefly, the names are composed of three parts, “ERV” and “DanRer” (Danio rerio) constitute the first and third parts, respectively. In this research, only the *Dr*ERVs related to class I–class III are initialed with “ERV”, whereas the others are initialed with “ERVL” (ERV-like). The second part indicates the locus. For example, in “ERV-E5.1.38-DanRer,” “5” represents chromosome 5, “1” represents the first 9 Mb fragments of chromosome 5, “38” represents the 38th *Dr*ERV in this fragment. In the *Dr*ERVs related to class I–class III, the second parts are initialed with “E” (epsilon-related), “AB” (alpha/beta-related), or “S” (spuma-related). Some *Dr*ERV genes that encode for Gag, Pol, and Env proteins are located beyond the expected *Dr*ERV regions (from 5′-LTR to 3′-LTR); these genes are annotated independently with additional “G,” “P,” or “E” at the end of the second part of the name, for example, “ERV-21.2.63E-DanRer.”

### Distribution analysis of *Dr*ERVs.

The correlation among element number, chromosome length, and GC content was analyzed using SPSS 17.0. χ^2^ test was performed using expected *Dr*ERV number per megabase (ratios of total *Dr*ERV number to total genome sequence length) and observed numbers per megabase (χ^2^ = ∑ [(observed − expected)^2^/expected]) to investigate the preference of *Dr*ERV distribution on chromosomes. The locations of NLR elements on chromosome 4 were indicated by the BLAST hits of FISNA, NACHT, LRR, and SPYR in the Pfam database (http://pfam.xfam.org/).

### Classification and phylogenetic analysis of *Dr*ERVs.

RT regions of *pol* genes were applied for the analysis as described previously (27, 40–43). Briefly, the RT regions were identified by Conserved Domain Database (CDD; https://www.ncbi.nlm.nih.gov/cdd/). The phylogenetic tree was constructed using DNA sequences of 968 predicted RT regions in *Dr*ERVs and 134 reference sequences from alpha-, beta-, delta-, gamma-, epsilon-, and spuma-retroviruses, and Gypsy, Copia, Bel elements, and ERVs from other species (Table S10). The reference sequences were retrieved from NCBI (https://www.ncbi.nlm.nih.gov/) and Repbase (https://www.girinst.org/). A *gag*-tree was constructed with DNA sequences of 454 retrotrans_gag domains of *gag* genes in *Dr*ERVs, as well as some XRVs and ERVs from other species. The XRVs and ERVs from other species were selected by BLAST search using retrotrans_gag domains in *Dr*ERVs, including NM_001040611.1, NM_001291326.1, XM_027102482.1, XM_029234152.1, XM_028119014.1, XM_021000084.1, XM_027848720.1, NM_001040152.2, EU726524.1, GU120138, NC_001452.1, and NC_001802.1. The *env*-tree was constructed with DNA sequences of TLV_coat/HR1-HR2 domains in 71 *env* genes, as well as some sequences selected by BLAST hits of these *env* genes, including DQ247958.1, XM_018224752.1, XM_016661903.1, NC_007654.1, XM_029730443.1, MG981046.1, XM_030132418.1, XM_031283908.2, XM_029056457.1, JX412978.1, NM_001305591.1, JN587107.1, XM_027598904.1, JN587101.1, NM_001305590.1, XM_030548363.1, XM_022686469.1, XM_028746725.1, KR049171.1, and XM_019290776.2. Sequences were aligned using MAFFT 7 with default settings (70), the high nonconserved sites were removed using trimAl (71). Maximum likelihood (ML) trees were constructed using IQTree with default settings (72), the best-fit sequence evolution model of GTR+F+G4 for RT tree, SYM+G4 for *gag* tree and TIM2e+I+G4 for *env* tree were suggested by ModelFinder (73), bootstrap 1,000 replicates was applied.

### Molecular dating.

The insertion time of *Dr*ERVs were estimated using the formula T = (D/R)/2, where T is the invasion time (million years), D is the 5′- and 3′-LTR divergence given as the number of differences per nucleotide per site (overall nucleotide divergence), and R is the genomic substitution rate per site per year. D was calculated using MEGA 6.06, the neutral rate of fish genomic evolution of 1.46 × 10^−8^ was applied (29).

### Characterization of *gag* and *env* genes.

The ORFs of *gag* and *env* genes were predicted by SeqBuilder in Lasergene 7.1 software (DNASTAR Inc., USA). Conserved domains in these ORFs of *gag* and *env* were further identified by CDD. Considering all retrotrans_gag-containing gag are Gypsy-like, we identified 55 ORFs at the *gag* loci in *Dr*ERVs without recognizable retrotrans_gag domains. The ML tree was constructed based on amino acid sequences of these ORFs as described in classification and phylogenetic analysis of *Dr*ERVs. The best-fit model of VT+I+G4 was applied. Additional sequences of XRVs and ERVs were selected by BLAST results, including EGV97139.1, AII72209.1, AAO62318.1, AGV92852.1, AAO46144.1, AAC78248.1, ADC92307.1, NC_043194.1, NC_001867.1, and NC_001724.1. For the *gag* genes without recognizable domains, we identified undiscovered conserved domains with MEME Suite (74), and these new domains were annotated by itol (https://itol.embl.de/). The ML tree of *env* genes constructed in classification and phylogenetic analysis of *Dr*ERVs was reused here for subsequent analysis. The conserved motifs in Env proteins were identified by comparing with the well-characterized Env proteins as reported previously (17, 18).

### Rapid amplification of cDNA ends.

Ten zebrafish were injected with 5 μL SVCV (10^7^ 50% tissue culture-infective dose [TCID_50_]/mL) per fish, and spleens were collected 12 hpi. Total RNAs were extracted using TRIzol Reagent (Invitrogen). The first strand was synthesized according to manufacturer’s protocol of the Single Cell Full Length mRNA-Amplification Kit (Vazyme, Nanjing). The ERV-E5.1.38-DanRer-*env* sequence was amplified by using specific primers and nested primers as shown in Table S11. The PCR products were purified from 1.2% agarose gel by using gel extraction kit (Omega) and inserted into pGEM-T EASY vector (Promega), then sequenced. Then, sequences were assembled manually and compared with genomic sequence.

### Comparative analysis between *Dr*ERVs and HERVs.

After removing the identical RTs, a total of 665 non-redundant RT sequences were used as queries to BLAST the human genome (GRCh38) in Ensembl (http://www.ensembl.org) database. The similarity between the queries and the hits were evaluated by scores and E-values provided by Ensembl. The RT sequences of ERV-E4.8.38-DanRer and HERV9NC-int were used to BLAST the genomes of platypus (mOrnAna1.p.v1), shark (Callorhinchus_milii-6.1.3), lizard (AnoCar2.0), mouse (GRCm39), duck (CAU_duck1.0), chicken (GRCg6a), koala (phaCin_unsw_v4.1), dog (CanFam3.1), and cow (ARS-UCD1.2) in Ensembl. The BLAST hits with highest similarity (evaluated by score and E-value, only hits with E-value <  1e-30 were selected) with ERV-E4.8.38-DanRer or HERV9NC-int were selected for phylogenetic analysis. A ML tree was constructed based on the amino acid sequences as described in Classification and phylogenetic analysis of *Dr*ERVs, with the best-fit model of LG+G4.

### RNA-seq analysis of *Dr*ERVs.

RNA-seq data of zebrafish embryo developmental stages was extracted from European Nucleotide Archive (PRJNA154389) (75). The data of Bud, 28 hpf, 2 dpf, and 5 dpf were selected for subsequent analysis. Tissues of heart, head kidney, spleen, gut, liver, brain, and muscle of healthy adult zebrafish were collected for subsequent experiment. Total RNAs were extracted using TRIzol Reagent (Invitrogen), and genomic DNA was removed using DNase I (TaKara). The spleen, head kidney, and gut tissues were collected 12 hpi with 5 μL SVCV (10^7^ TCID_50_/mL) or mock PBS per fish, and RNAs were extracted as mentioned above. RNA-seq transcriptome libraries were prepared following TruSeq RNA sample preparation Kit from Illumina (San Diego, CA), and mRNAs was isolated by oligo(dT) beads and then fragmented by fragmentation buffer. Double-stranded cDNA was synthesized using a SuperScript double-stranded cDNA synthesis kit (Invitrogen, CA, USA) with random hexamer primers (Illumina). Libraries were sequenced with the Illumina NovaSeq 6000 sequencer (2 × 150 bp read length). Then, reads were mapped to zebrafish reference genome (GRCz11) using TopHat; and quality control was performed using RseQC. The pattern recognition and clustering analyses were performed using scripts of R; differential expression was analyzed using DESeq, scripts of R and perl. Transacting analysis for ERV-like elements was performed using R script. The Pearson correlation coefficient and *P* value of expression level of ERV-like elements and mRNAs were assessed, and absolute value of Pearson correlation coefficient greater than 0.85 and *P* value less than 0.01 is considered transacting. GO enrichment was performed using the R topGO package. The mapping between Ensembl IDs and GO terms was retrieved from the Ensembl database using a custom Perl script (get_ensembl_go_terms.pl) from the topgo-wrapper repository (https://github.com/iansealy/topgo-wrapper).

### Molecular cloning.

The primers for zebrafish IRF1 and RelA were designed according to sequences in Ensembl (http://www.ensembl.org). Total RNAs were extracted from zebrafish spleen by using TRIzol Reagent (Invitrogen) and reverse-transcribed into cDNAs, then IRF1 and RelA cDNAs were generated through RT-PCR. The PCR products were purified from 1.2% agarose gel by using gel extraction kit (Omega), and inserted into the pcDNA6 (Invitrogen) and pEGFP-N1 (BD Biosciences) vectors. The plasmids were transformed into competent Escherichia coli DH5α (Invitrogen), and the positive plasmids were purified by using endo-free plasmid minikit II (Omega Bio-tek).

### Quantitative RT-PCR for expression analysis.

The transcriptional expression of *fga*, *igl1c3*, ERVL-7.2.48-DanRer, and ERVL-3.1.72-DanRer upon SVCV infection were determined by quantitative RT-PCR (Q-RT-PCR) on a Mastercycler ep realplex machine (Eppendorf). SVCV infection and RNA preparation were performed as described in RACE, and RNAs were reverse-transcribed into cDNAs. PCR experiments were performed in a total volume of 10 μL by using a SYBR Premix *Ex Taq* kit (TaKaRa Bio). The reaction mixtures were incubated for 2 min at 95°C, then subjected to 40 cycles of 15 s at 95°C, 15 s at 60°C, and 20 s at 72°C. The relative expression levels were calculated using the 2^−ΔCt^ and 2^−ΔΔCt^ method with β-actin for normalization. Each PCR trial was run in triplicate parallel reactions and repeated three times.

### Chromatin immunoprecipitation-qPCR.

Promoters were predicted for *Dr*ERVs surrounding immune-related genes with PROMO (76, 77). ChIP assays were applied to investigate whether *Dr*ERVs would serve as transcriptional factor binding sites for downstream genes. Zebrafish embryos were acquired as described above. Plasmids of pcDNA6-IRF1 and pEGFP-RelA were microinjected into the embryos with amount of 200 ng/embryo at 0.5 hpf, followed by injection of 2 nL PBS or SVCV (10^7^ TCID_50_/mL) per embryo at 24 hpf. At 36 hpf, 30 embryos were collected for each replicate in both PBS and SVCV administered groups (three replicates in each group). ChIP assays were carried out according to the manufacturer’s protocol of the ChIP assay kit (Beyotime, Beijing). Samples with normal mice IgG and the input were used as negative and positive controls. Pull-down levels of target promoter sequences were determined by qPCR and normalized to the corresponding abundance in the input chromatin. The promoter-specific primers of indicated *Dr*ERVs are listed in Table S11.

### Statistical analysis.

Statistical analysis and graphical presentation were carried out using SPSS 21.0 and GraphPad Prism 6.0. Quantitative data from the three independent experiments were expressed as mean ± SD. The groups were compared statistically using Student's *t* test for paired samples. The *P* values *, *P* < 0.05, **, *P* < 0.01, and ***, *P* < 0.001 were considered statistically significant.

### Data availability.

Raw data of RNA-Seq have been deposited into (Sequence Read Archive (SRA)), with the accession number PRJNA690124 (for expression profile of 7 tissues) and PRJNA690234 (for expression profile in response to SVCV infection).
