# Supplementary material for: Genome-Wide Characterization of Zebrafish Endogenous Retroviruses Reveals Unexpected Diversity in Genetic Organizations and Functional Potentials
Source: Microbiol Spectr. 2021 Dec 15;9(3):e02254-21. doi: 10.1128/spectrum.02254-21 (PMC8672886; doi:10.1128/spectrum.02254-21)
Supplement: SUPPLEMENTAL FILE 2 — Supplemental material. Download SPECTRUM02254-21_Supp_1_seq3.pdf, PDF file, 3.3 MB [file spectrum02254-21_supp_1_seq3.pdf]

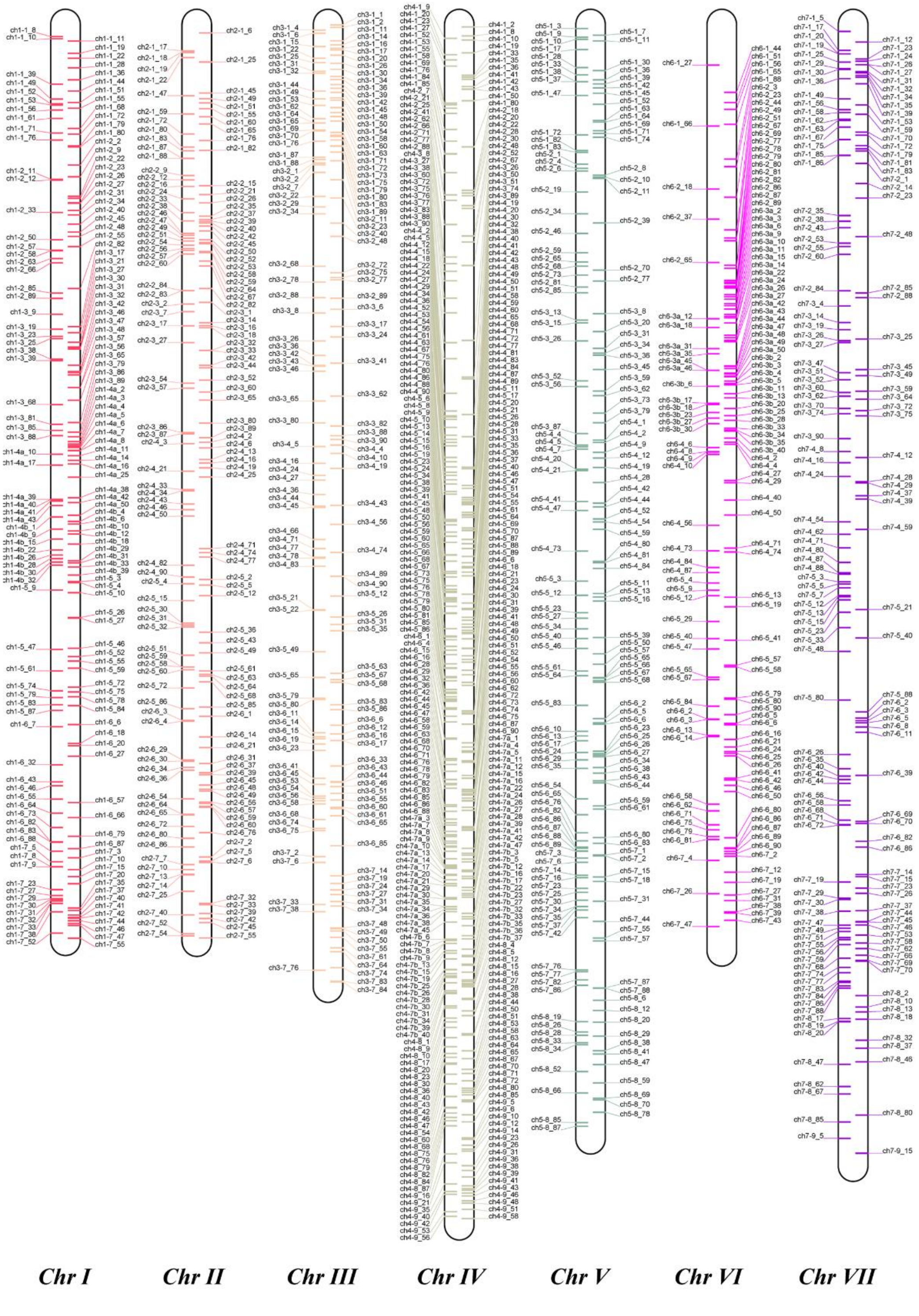

Figure S1. Localization of *Dr*ERVs in zebrafish chromosome 1–7.

The abbreviations of the names of *Dr*ERVs are used

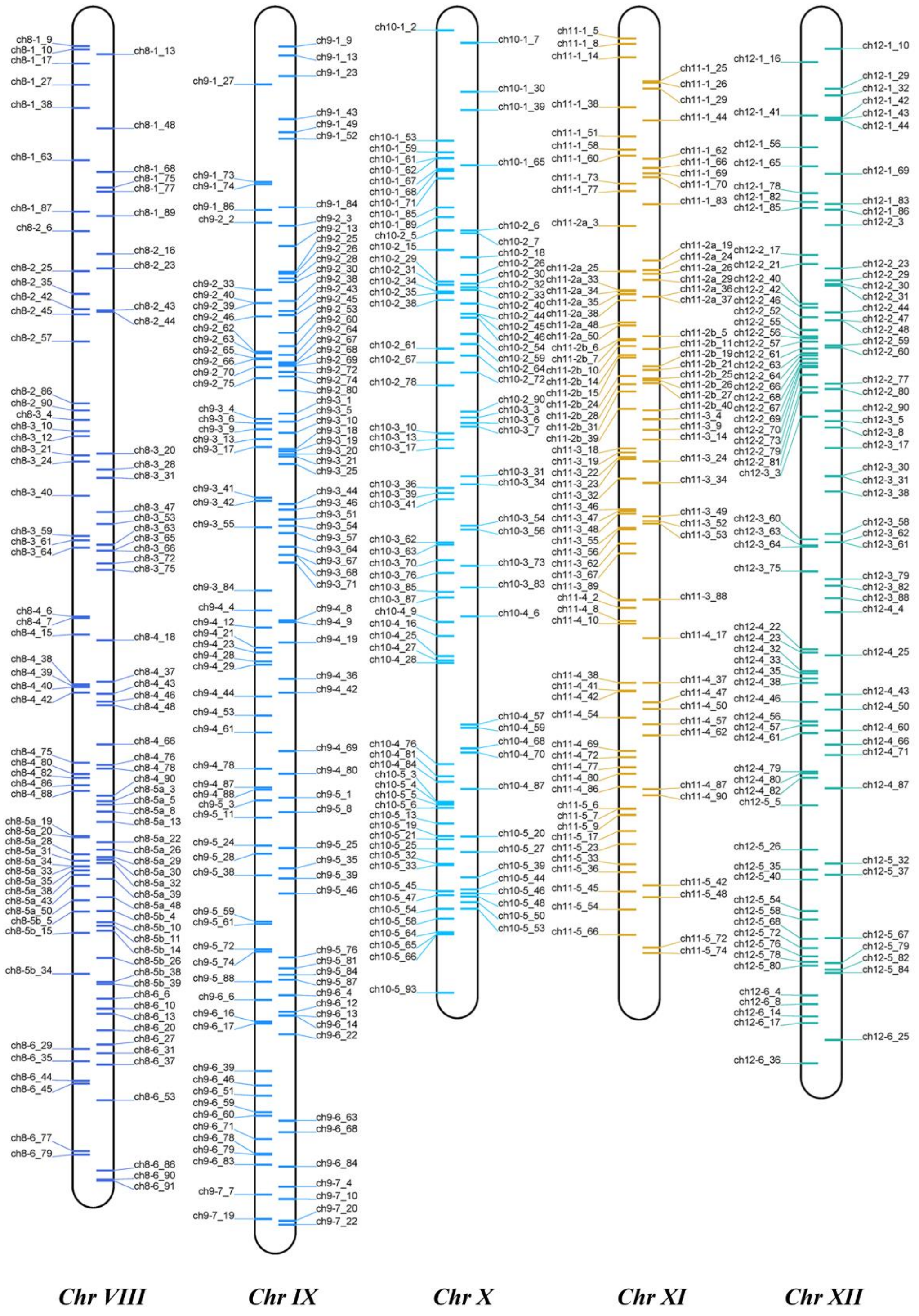

Figure S2. Localization of *Dr*ERVs in zebrafish chromosome 8–12

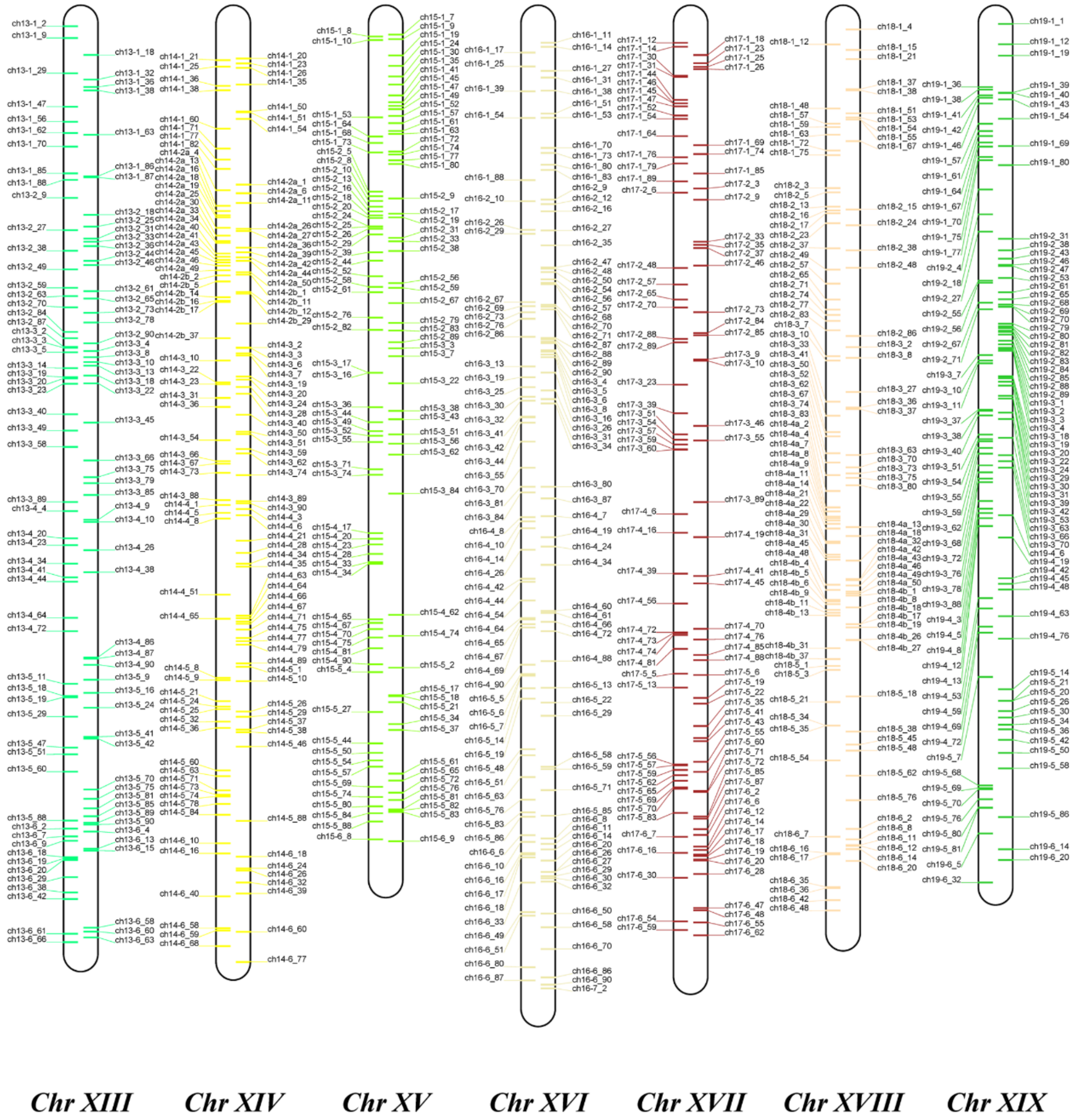

Figure S3. Localization of *Dr*ERVs in zebrafish chromosome 13–19

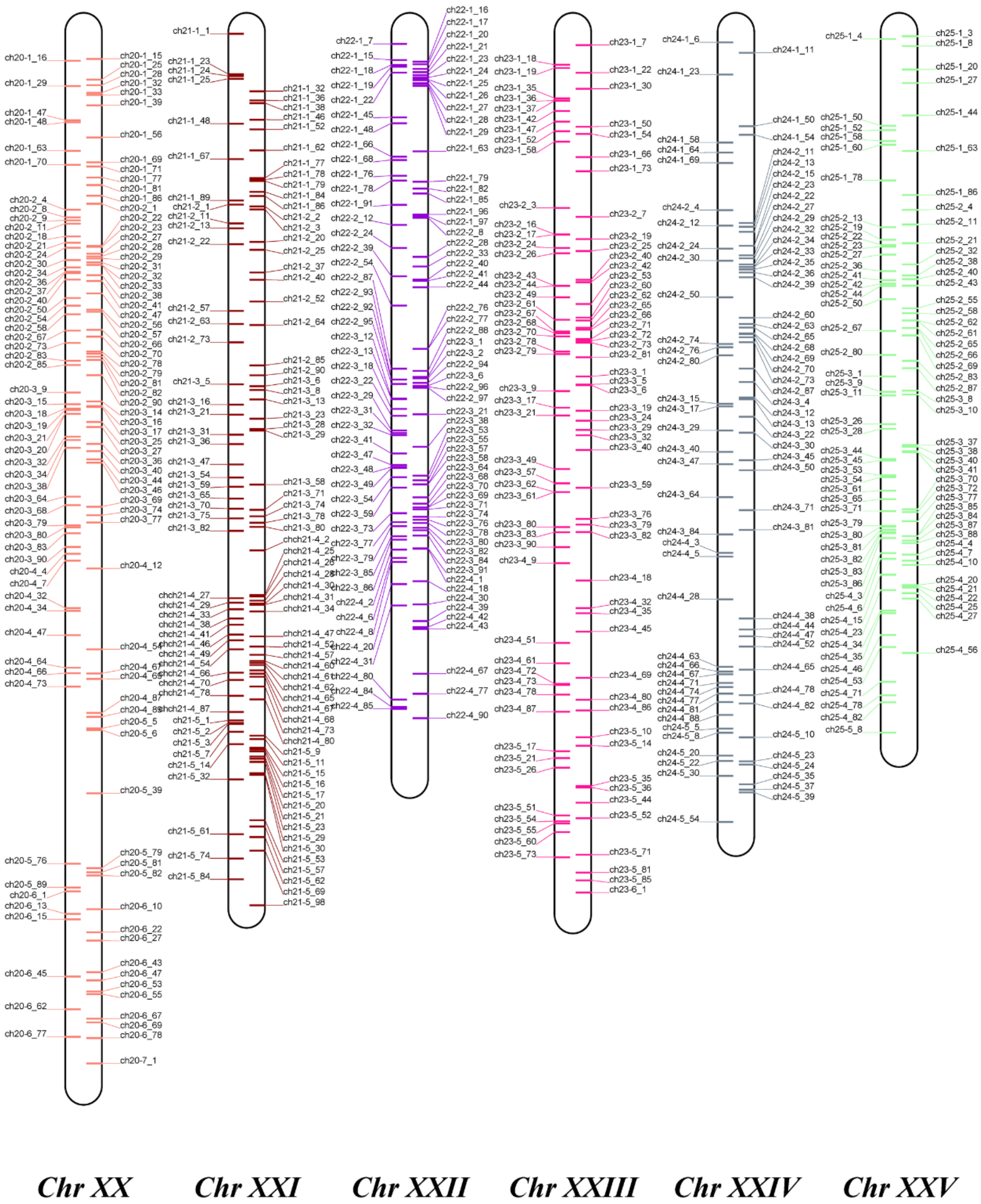

Figure S4. Localization of *Dr*ERVs in zebrafish chromosome 20–25

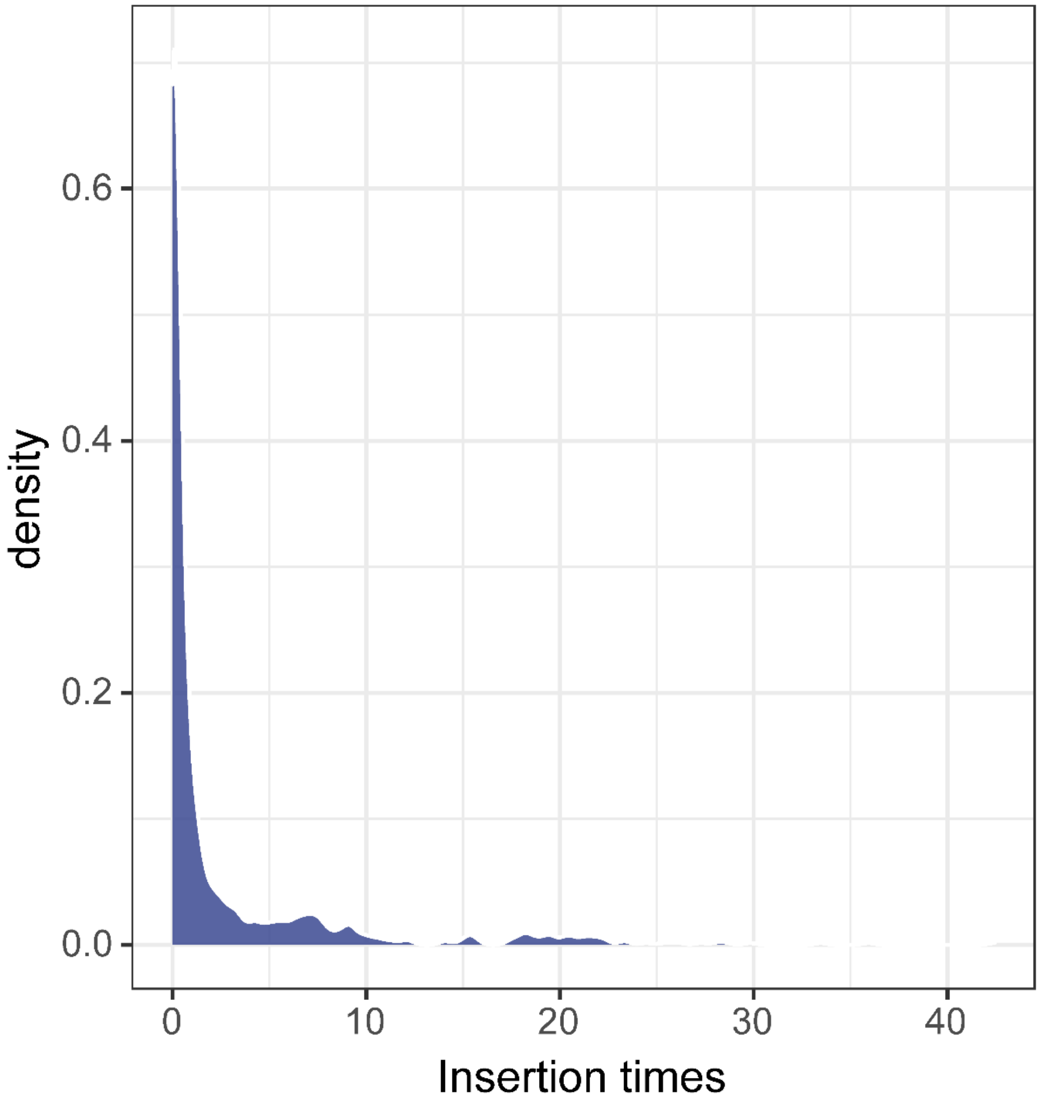

Figure S5. Density distribution of the insertion times of *Dr*ERVs

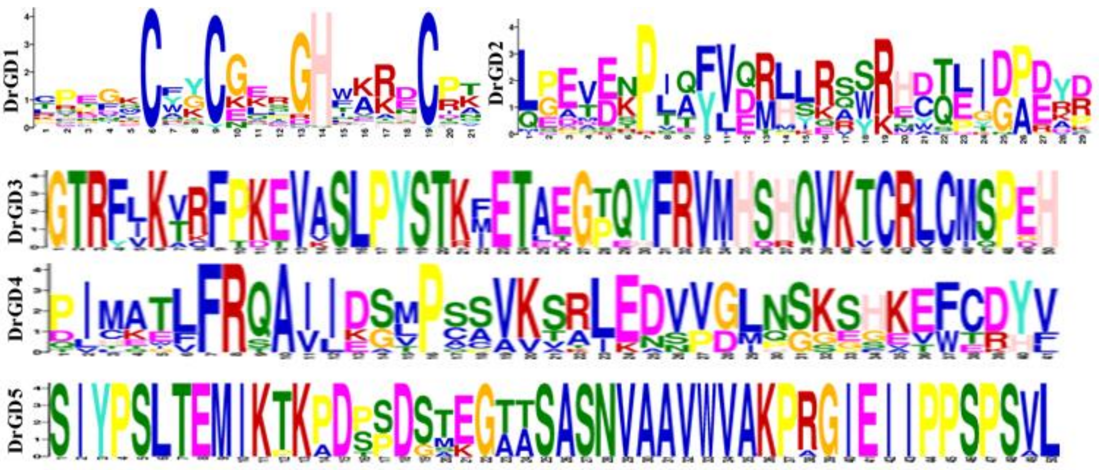

Figure S6. Consensus sequences of the predicted DrGD1-5 domains in *gag* genes

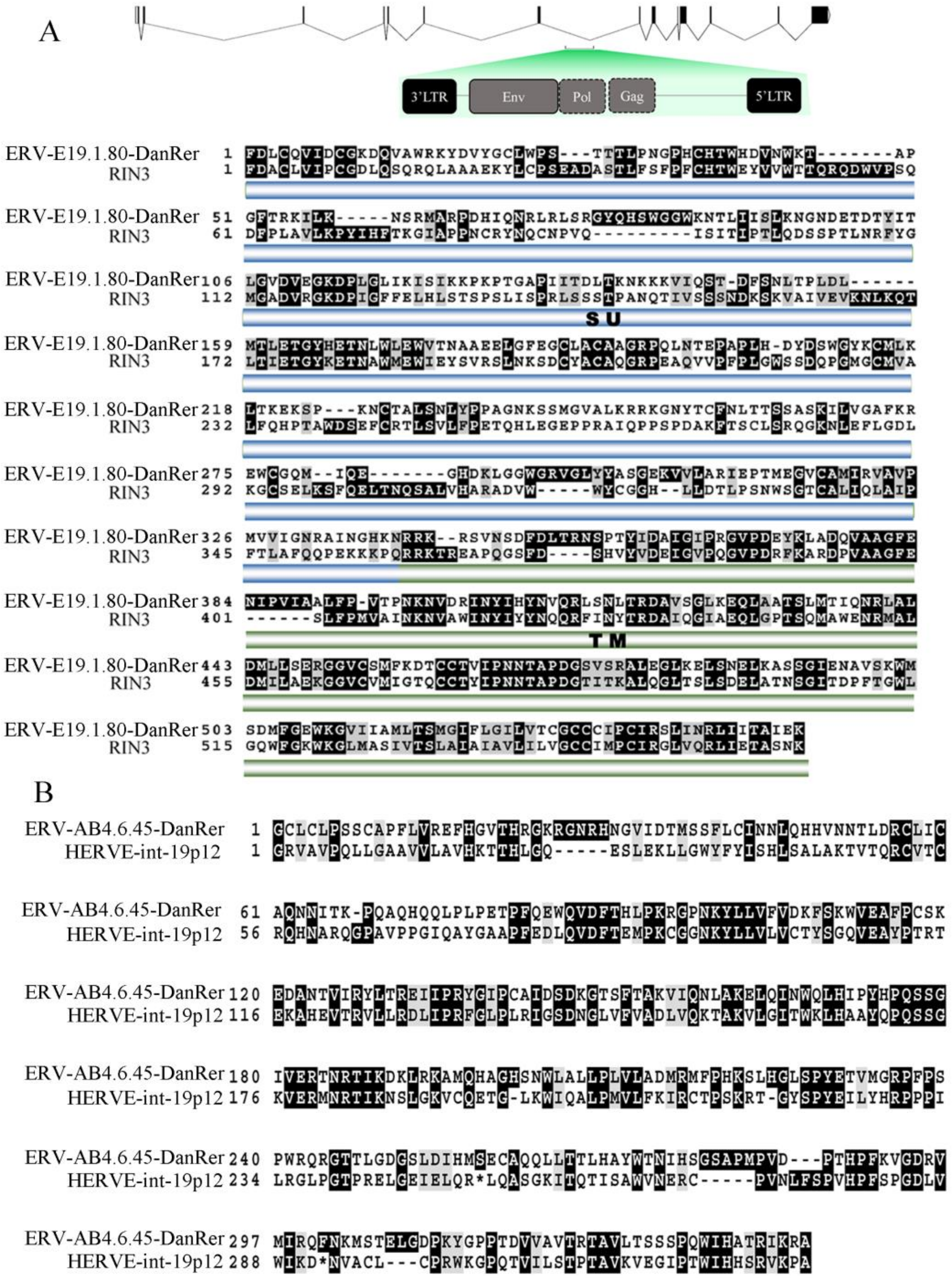

Figure S7. Sequence comparison of Env and Gag between *Dr*ERVs and HERVs

(A) Amino-acid sequence alignment of ERV-E19.1.80-DanRer-*env* with the *env*-like element in intron of human RIN3, in which the putative SU domain is marked up by blue line, and the TM domain is marked up by green line. (B) Amino-acid sequence alignment of ERV-AB4.6.45-DanRer-*gag* with that of the HERVE-int-19p12.

> HERV-14q32.12

CGTTGTTAAGAACAAATAGTTCCTCTTCAAAGGGTTTTGGTTCCTGGTTCTTTGTTCTATTCTAAAAGGTAATCTTACCCATTCATCTATTAGCCCT
CCCTATCTCCACTGTGCCTAAACACACCCAAGATATACTGTACCTGTTCTCCCTGTCAAACGCCCACCCTCGCCTCCTTTGATGATGTCAACAGTA
GCCAATCGGAATTAGCCTAGATTGTGTGGTCCGACCCCAGCCCACAGGGGGAGGACACAGGAACAGGGTCTGCTTCAGGGATAAAAACTCCTG
GTCTCCTTTGTTTTGCAAGCTCTCCCTTGCCTCTGCTTTGCATGCAGGAAGCGCCCTTCTATAGAAGTAAATTGCCTTGCTGAGAAAACTTTTGCC
TGAATGCTGGGTTTCACTTCACAGCACTGACAATTTGTTTCTAACAATCTGGGGCCTTGCCCAGGATTCCCATTCTCCTCTGGGAAGGGGTCTC
TGGTCATCTCCCAGGGGGAGACGTGACCCACTGCCTTGCTGACGTGGCCTCAAGGGCTAGAGATTGGGACCCCACCCACTGTGACGAATAAAC
CTGGACTCTCGGCAACGTTGGAAGGAAAGACTTGCCTACAAATACCGTGGCGACCAGGTAACTTTGCGCACAAGACCAAAGTAAGAAAAGCTG
TGGGGGCGGTGAAGTATTTCATTGGTGGTCGGGATATCCTGGAGGTTGAAAGTGTGTGTGAATGATCATAGGCACCACTGCTTGCGGTGCTGCTT
GTGTGAATGGTGATAGGCATTACTGCTGTCCGGGGTGAGTGGGTCCTGTCTGTGGTTCTGTGGTTCTGTGGTCACCTCATCTTCATATGGCTTAGG
GCAGATCCTGCCATGGGGCTTATACCTGCATGCCAATGCTAAGAGGGACCTAATTTCCTGTGAAGGAAGTGGCCAGATAGGATGAAGTGAATGGA
AAGGAGTGCAAGAAACCTCCATTAATGGGGGTTGAGCCTCTAGAGAAAAGGGAAGGCAAGAGAACTCTAGTAAGAGAGATTGGGCCACACAC
ACAGTCAAGGGGGCATCCAAAACTTCCAAGATGGGAAACACTCCTAGTACAACAGGGAAAGGTAAGGACGGGGATAGTCAGATCCCCTCTGAT
AGTCCTCTAGGTCTCATGCTGAAATATTAGAAGGAGAATGAGAGGAATAGGCACAAGAAAAAGCAACAGATGATAAAATATTGTTGTTTCATTTG
GGCCAAGGAACCCATCCGCAAACCTGCCATCTTCTTGCCAAAATATGGATCAGATGAGGATTGGGTCTGTTAACTTTTAACTCAGTATGTGAATG
ACAAAAGTCTGGTCTTGCGGGAAGAGACTATGCCCTCTTCTGGAGAAAACAACCTGTTCTCCTCTTTCCCCTAAAGAATACAGGGGAAGGCGTA
GTAAAACCAACCCCTAGGCAGAGGGATCCCAACTCCTGGAACCCTCTAAACCACCTTTCCCCATTTAATTCCCCCCAACTTGACCTGTCCCCTCC
ACAGGCAGCTGCTGCTACCCTGGACTCGGTCCCAGATCCTTCTGCTACTCCTATTATTCCTCCCCCTTATACTCTGACTCTTGGGAATTACCACCAC
CCCATGAGTCTGTTCCTTGTCAGCCTAAGTATCCTTCCCTAAAGGGACTACCATGTAAGAAGGATATTCAAAATCTTCCTTTCCCCTCTGCCTCTAA
GGAATCAGCTCCAACTCTCTTCCCTTTGAGAGAGGTGTCCCAGGCAGGAGGAGCTACTGGCTTTGTAAATGCTCCCTTGACTAGTTCAGAGGTT
CAAGGTTTAAAGAAAGAACTTAAGCCACTGCTAGATGACCCATATGGGGTAGCAGATCAATTCCTAGGCCCCCAGTTATATACCTGGGTCGAATT
AATGTCCATCCTGGGTATCCTCTTCTCAGGGGAAGAAAGGGGGATGATTCACAGGGCTGCTATGACAATTTGGGAATGTGAACATTCTCCTGGCC
AAAATGTTCCTGCAGCAGACCAGAAATTCCCAGCCCAAGATCCTCAATGGGATAATAACAATGCAGTCTGCTGGGAAAATATGAAAGATCTTAGG
GAAATGATAATTAAAGGAATTCGGGAGTCAGTGCCCCATGCTCAGAATCTTTCCCGAACATTTGACATACAACAGGAAAAAGATGAAGGGCCCA
TGAAATTTCTAGAAAGACTAAGGGAACAAATAAGGAAATATGCAGATTTGGATCCAGAAGACCCTCTTGGGCAAGGAATATTAAAGCTACATTTT
GTCACAAATAGCTGGCCAGATATAGCAAAGAAGTTGCAGAAACTAGAAAAACAAAAAACCGGTCCAGAGAGGAACCCCTAGGAGAAGCCCAA
AAAGTATATGTGAGGAGAGATTAAGAAAAGCAAAAACAAAAAATGAAACTTATGCTATCCACCTTCCAACAGACGGCTTCAAGCCATATGCTTC
TAAACAGAGACTCCAGGGGACCAGGAATTATAAAGGGTCCAAACCCAAGCCTCCAACTAGCGGACCCAGACCCTCAGCTACCAGGCCCTCTAA
AGAGTATGGGGGAGCAAGGTCAAATAATCCTGGAAATGAGAGAGTGGGAGGACAGGATAGGTGCTTCAAATATGGAAGAGCAGGCCACTTCAA
AAGAGAATGCCCTGAGTTGGGGAAGGAAAAAGAAACTGTCCTGCTCATGGCATAATAGTAGACCACCTCACCCATTGAGTTGAAGCCATTCCCC
TCCCCGCAACAGCTAATAATGTGGTTAAAGTATTACTAGAGAAGATAATACCTCAGTTCGGGCCAGTAGAAAACACTGACTCAGACAACAGGAAT
CACTTTACCGCAAATATCATTAAAGGGCTTACTCAAACTTTAGGAATCAAATGGGAGTATCATGACCCCTGGCATCCATCCTCATCAGGAAGAATA
GAAAGGATGAATCAGACTTTAAAAAATCATCTAAGTTAATTTCAGAAAGTCCATTACCCTGGACTAAGTGTCTCCCCATCGCTCTACTCAGGATCC
GAACTGCCCCTCAAACAGATGTTGGCCTATCCCCTTATGAAATGCTCTATAGGTTACCATACTTAAGTTCCACTAGTGACAGTCCCACTTTTGAGA
CCAAAGATCAGTTCCTTAGGAATTATATACTTGGTCTGTCTTCTACCTTATCTTCCCTTAGGACTAAAGGTCTCTTAGCGCAAACGCTGCCTCTTGA
GTTTCCAGTCCATCAGCACCGGCCCGGAGACTACGGCCTCAATAAGAGCTGGAAAGAAGAGAAACTTGAACCGTCCTGGGAGGGACCATATCT
AGTGCTCCTAACAACTGAAACTGCAGTCTGAACTGCCGAGAAAGGATGGACCCATCACACCCGAGTCAAGAAAGCACCGCCCCCTCCAGCGTC
ATGGGCCATAGTCCCAGGGGAAAATCCTACCAAACTAAAGCTAAGGAAAGTTTAATTCTCTTTCATCTATTCTGTTACTCCTTCTTCTTTCCTTGTG
CTCTTGCTAGCCACCTCATTATTAACGTAACTAGATCAGACTCACCCCAGACCATTACCTTCGATGCTTGTTTAGTTATACCTTGTGGGGATCTCCA
AAGCCAGAGACAGCTTGCAGCAGCAGAGAAATATCTCTGCCCCTCCGAAGCAGATGCTTCTACCTTGTTTAGCTTTCCGTTTTGTCATACTTGGG
AATATGTCGTTTGGACCACTCAACGTCAAGATTGGGTCCCCTCACAGGATTTCCCGCTAGCGGTTCTAAAGCCCTATATCCATTTTACTAAAGGAA
TTGCCCCTCCCAATTGTCGATATAACCAATGTAACCCGGTGCAAATTTCCATCACCATCCCAACTCTCCAAGATTCCTCCCCCACCCTAAACCGTT
TCTATGGTATGGGAGCAGATGTAAGAGGGAAAGACCCCATAGGATTCTTCGAGTTGCACCTCAGTACATCTCCATCCCTCATATCTCCACGTCTAT
CCTCTTCTACACCTGCTAACCAGACCATTGTCTCTTCATCTAATGACAAAAGCAAAGTAGCTATTGTAGAGGTTAAAAATTTAAAACAAACACTG
ACAATTGAAACAGGATACAAAGAAACAAATGCCTGGATGGAATGGATTGAATATTCCGTTCGCAGTCTAAACAAAAGCGACTGTTACGCTTGTG
CGCAAGGTAGGCCAGAGGCCCAAGTCGTCCCCTTTCCACTTGGATGGTCTTCTGACCAACCGGGCATGGGCTGCATGGTGGCTCTTTTCCAACA
CCCTACAGCCTGGGATAGTGAATTCTGTCGAACTCTCTCTGTGCTATTCCCTGAAACTCAACACCTTGAGGGTGAGCCCCCGAGGGCCATCCAGC
CTCCATCTCCAGATGCCAAGTTTACTTCCTGTCTCTCACGACAGGGAAAAAATTTGGAGTTTCTTGGGGACCTAAAGGGATGCAGTGAGCTTAAG
TCTTTCCAAGAGCTTACCAATCAGTCTGCACTTGTTCATGCCCGAGCTGATGTATGGTGGTATTGTGGTGGTCACCTGCTAGACACTCTGCCAAGT
AACTGGAGTGGTACTTGTGCTCTAATTCAATTGGCCATCCCTTTCACCCTGGCATTTCAACAACCAGAAAAAAAGAAACCACAACGCCGTAAAA
CAAGAGAGGCCCCTCAAGGATCTTTCGACTCTCATGTCTATGTAGATGAAATTGGAGTCCCACAGGGGGTACCTGATAGATTCAAAGCCCGAGA
CCCAGTAGCTGCAGGATTTGAATCATTATTTCCAATGGTAGCTATTAATAAAAATGTAGCTTGGATAAATTACATCTATTATAACCAGCAGCGATTTA
TTAATTACACTAGGGATGCTATCCAAGGAATAGCTGAACAATTAGGGCCTACTAGCCAAATGGCTTGGGAAAACAGAATGGCCTTAGATATGATAT
TAGCAGAAAAAGGTGGAGTTTGTGTTATGATAGGAACCCAATGCTGCACCTACATTCCCAACAATACAGCTCCTGATGGAACAATTACAAAAGCT
TTACAAGGTCTTACCTCCTTATCAGATGAATTAGCCACAAATTCTGGGATAACTGACCCTTTCACAGGATGGTTAGGGCAATGGTTTGGTAAATGG
AAAGGACTCATGGCCTCTATTGTTACCTCTCTCGCAATCGCAATAGCTGTGCTTATTCTTGTTGGATGCTGCATCATGCCCTGCATTCGTGGACTAG
TCCAAAGACTTATAGAGACAGCTAGTAACAAAACCTTCCCTAGTTCTTCCCAATCCTATAGTAACAAATTCTTCCCCGTGAACGAACACGAAATC
CGAATCATATTAGATAGGTTTAAAGCAGAACATGTATAAATTCAAGAGGAGGAAATTGTTGTTGACCACAAATAGTTCCTCTTCAAAGAGTTTCTG
TGCCTGGTTCTTTGTTCTATTCTAAAAGGTAATCATACCCATTCATCTATCAGCCCTCCCTCTCTCTGCTGTGCCCAAACACCCCAAAATGTACTAT
ACCTGTGCTTCCTGTCAAAGGCCCACCCTTGCCTCCTTTGATGATGTCAACTGTAGACAATCGGAATTAGCCTAGATTGTGCGGTCTGACCCCAG
CCCACAGGGGGAAGACACAGGAACAGGGTCTGCGTTAGGGATAAGAAAATCCTGTTCTCCTTTGTTTTGCATGCTCCCCCTTGCCTCTGCCTTGC
ATGCAGGAAGCGCCCTTCTACAGAAGTAAATTGCCTTGCTGAGAAAACTTTTGCCTGAGTGCTGGGTTCACTTTGTGGCACTGAAAATTTGTTTC
TAACA

LTR Gag Pol Env LTR

Figure S8. Sequences and annotation of HERV-14q32.12

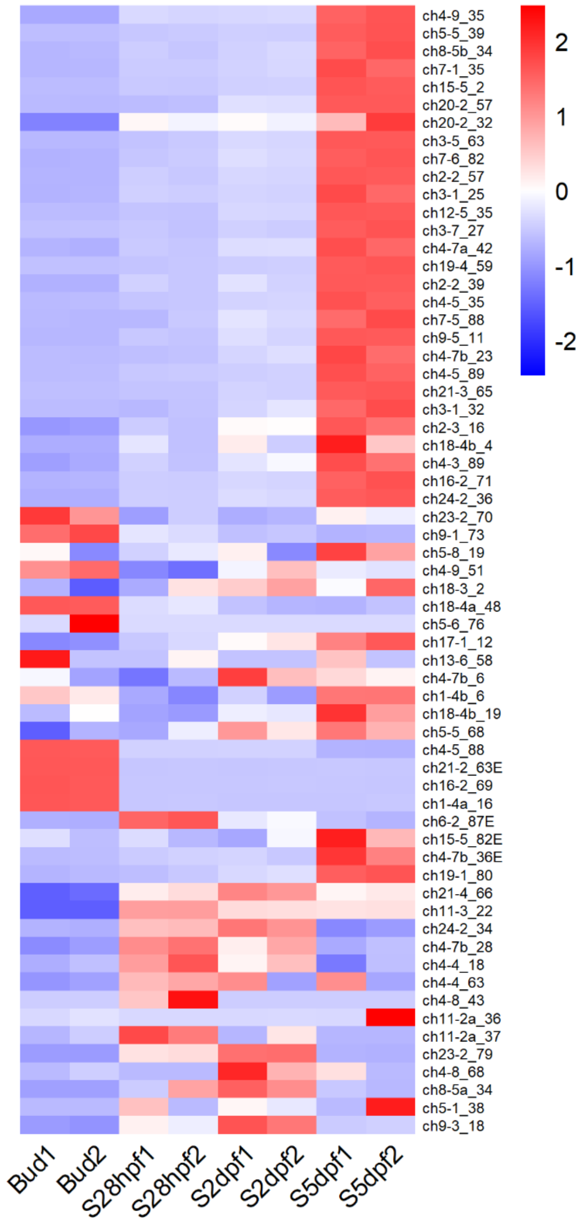

Figure S9. Expression of *env*1-4 at 4 embryonic stages.

The abbreviations of the names of *Dr*ERVs are used

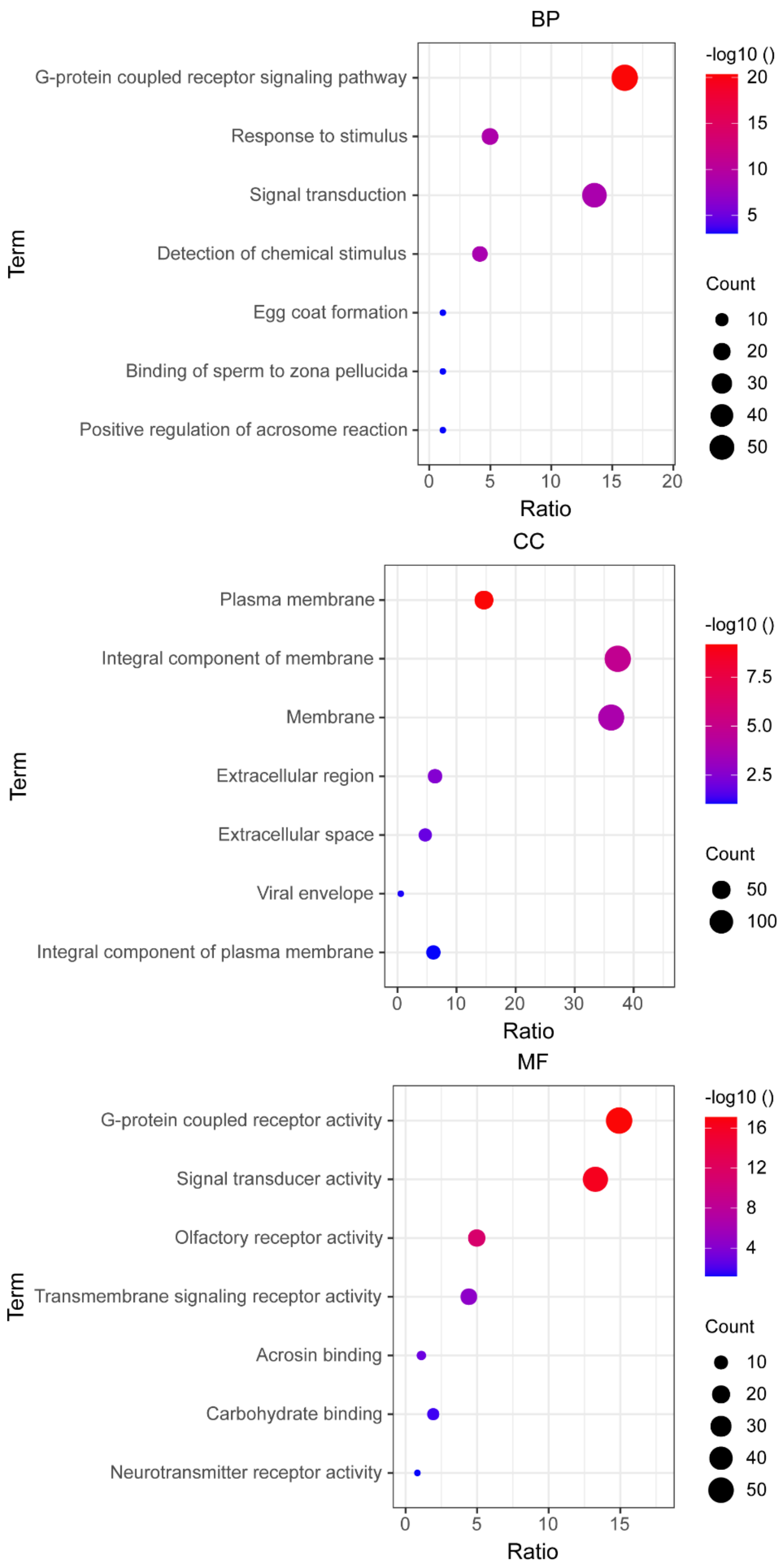

Figure S10. GO enrichment of genes co-expressed with *Dr*ERV upon SVCV infection
